# Natural compounds as safe therapeutic options for ulcerative colitis

**DOI:** 10.1007/s10787-022-00931-1

**Published:** 2022-02-25

**Authors:** Mukta Gupta, Vijay Mishra, Monica Gulati, Bhupinder Kapoor, Amrinder Kaur, Reena Gupta, Murtaza M. Tambuwala

**Affiliations:** 1 School of Pharmaceutical Sciences, Lovely Professional University, Phagwara, Punjab 144411 India; 2 School of Pharmacy and Pharmaceutical Sciences, Ulster University, Coleraine, BT52 1SA Northern Ireland, UK

**Keywords:** Ulcerative colitis, Herbal constituents, Anti-ulcerogenic activity, Inflammatory bowel disease

## Abstract

Ulcerative colitis (UC) is a chronic inflammatory bowel disease of unknown etiology. Several conventional treatments for UC such as corticosteroids, immunosuppressive agents, tumor necrosis factor antagonist, integrin blockers, and interleukin antagonist, and salicylates are available but are associated with the various limitations and side-effects. None of the above treatments helps to achieve the ultimate goal of the therapy, i.e., maintenance of remission in the long-term. Natural remedies for the treatment of UC show comparatively less side effects as compared to conventional approaches, and affordable. The current review presents details on the role of herbal drugs in the treatment and cure of UC. Google, PubMed, Web of Science, and Scopus portals have been searched for potentially relevant literature to get the latest developments and updated information related to use of natural drugs in the treatment of UC. Natural products have been used over centuries to treat UC. Some of the essential herbal constituents exhibiting antiulcerogenic activity include gymnemic acid (*Gymnema sylvestre*), shagoal (*Zingiber officinale*), catechin (*Camellia sinensis*), curcumin (*Curcuma longa*), arctigenin (*Arctium lappa*), and boswellic acid (*Boswellia serrata*). Although many plant-derived products have been recommended for UC, further research to understand the exact molecular mechanism is still warranted to establish their usefulness clinically.

## Introduction

According to the World Health Organization (WHO) report, more than 80% of the world’s population relies on the traditional system of medicine for their health problems (World Health Organization 2019). Traditional medicines, mainly herbal products, serve as a lead compounds for identifying other bioactives as these have been used for thousands of years for treating various types of diseases and have the advantages of lower side effects, better availability and cost effectiveness (Choi et al. 2016; Huang et al. 2010; Lin et al. 2014). As the prevalence of chronic diseases, including cardiovascular system disorders, diabetes, cancer, ulcerative colitis (UC), and acquired immunodeficiency syndrome (AIDS) is increasing day by day; herbal medicines have gained popularity in the healthcare system and have been recommended to be used globally for these diseases. Moreover, several clinical and pre-clinical studies have been conducted for evaluation of the effectiveness and safety of such herbal remedies (Choi et al. 2016; Quansah and Karikari 2016). The UC, a type of inflammatory bowel disease (IBD), generally affects the mucosal lining of colon resulting in inflammation and ulcers.

## Epidemiology

The IBD is a collective term used for a group of chronic manifestations that affect the small and large intestine and is a common cause of gastrointestinal morbidity (Fruet et al. 2012; Zois et al. 2010). The risk factors for IBD involve the overproduction of free radicals and decreased antioxidant capacity (Aleisa et al. 2014; Parfenov 2012).

The two primary forms of IBD are UC and Crohn’s disease (CD). According to WHO, the prevalence of UC is estimated to be 200–250 per 100,000. It is more common in western countries and is increasing worldwide (Annaházi and Molnár 2014; Campbell et al. 2001; Porter et al. 2020). UC affects both sexes equally and can start at any age; however, the primary age of onset of the disease is 15–30 years (Annaházi and Molnár 2014).

## Etiology

The exact cause of UC is not known to date. It is multifaceted disorder where genetic factors, infective agents,  oxidative stress, dysfunction of immune regulation, overproduction of prostaglandin (PG) E2 and the loss of tolerance of the luminal microbiota are key contributors to the development of this disease (Awaad et al. 2013; De Almeida et al. 2013; Fruet et al. 2012; Zhang et al. 2006). Among all, oxidative stress contributes the most, in which interplay between reactive oxygen species (ROS) and reactive nitrogen species (RNS) is responsible for many physiological functions and colorectal pathological processes. Therefore, there has been an increase in interest in the potential uses of exogenous antioxidants to treat and prevent oxidative gastrointestinal disorders (Aleisa et al. 2014). UC is also initiated and promoted by release of inflammatory cytokines by macrophages, B-cells, and T-cells. Various pro-inflammatory cytokines involved in articular cartilage destruction are tumor necrosis factor-α (TNF-α), interleukin-1 (IL-1), IL-6, IL-8, granulocyte–macrophage colony-stimulating factor, and transforming growth factor-β) (TGF-β) (Patil and Moss 2008; Toshifumi 2003; Clinton 2009).

## Symptoms of ulcerative colitis

The UC exhibits many characteristic features like chronic remitting, relapsing course, inflammatory nature, and unknown causes (Bamias et al. 2005; Hirten and Sands 2021; Samanta et al. 2012; Hendrickson et al. 2002). Some other symptoms are fatigue, tiredness, fever, nausea, diarrhea, bloody stool, anorexia, weight loss, malaise, delayed growth, arthritis, and sometimes anemia (Sninsky 2010). Although the transformation of UC to CD is not frequent, the pathological finding performed during clinical studies confirmed the first case of the progress of UC to CD (Satish Chandra Yadav 2021).

## Available treatment approaches for ulcerative colitis

The ultimate goals of currently used antiulcerogenic drugs are not only to control disease progression but also to induce a quick remission and to maintain it for a long time along while preventing complications of the disease itself, minimize disability, and hence improving patient life and expectancy (Annaházi and Molnár 2014; Hanauer 2008; Probert et al. 2014). The choice of therapy depends on the severity of the condition, i.e., the extent of colon involvement and its localization. Further treatment depends upon the primary response of induction therapy (Meier and Sturm 2011; Theede et al. 2013; Sharma and Mishra 2014).

Therapy for UC consists of the following two steps: the first-line treatment is to induce remission (with induction agents) and resolve all inflammatory symptoms while the second is to maintain remission (with maintenance agents) (Nanda and Moss 2012; Dalal 2007). Most of these objectives are achieved by the combination of salicylates (like mesalazine and olsalazine); immunomodulators (like azathioprine, 6-mercaptopurine, cyclosporine, and methotrexate); corticosteroids (like methylprednisolone, and prednisolone); tumor necrosis factor signalling inhibitor (like infliximab, adalimumab and golizumab); integrin blocker (like vedolizumab, natalizumab etrolizumab); Janus kinase (JAK) inhibitor (like tofacitinib); and interleukin antagonist (like mirikizumab and ustekinumab) (Sands et al. 2014; Witaicenis et al. 2012). Along with the same, colectomy (surgical treatment) may be an alternative choice in case of life-threatening complications. The potential therapeutic agents for the treatment of UC and their targets are tabulated in Table 1. In addition to conventional therapies, some unconventional treatments, including leukocytapheresis, inorganic nitrite or nitrate, and fecal bacteriotherapy, have been explored to treat UC (Yokoyama et al. 2014; Jädert et al. 2014; Borody et al. 2003).

**Table 1:** Therapeutic agents used in UC and their complications

Pharmacological class/treatment	Drugs	Target	Complication	References
5-Aminosalicylates	Sulfasalazine, mesalamine/mesalazine, olsalazine, and balsalazide	COX, IL-1, TNF-α, LOX, NF-κB, PPAR-γ	Headache, diarrhea, cramps, abdominal pain and renal impairment	Biancone et al. (2008), Caprilli et al. (2009), Carter et al. (2004), Chapman and Rubin (2014), Dalal (2007), Nanda and Moss (2012)
Corticosteroids	Budesonide, hydrocortisone, methylprednisolone, and prednisone	Immune system modulator, IL-1β, TNF-α, MMP-9	Hyperglycaemia, hypertension, electrolyte disturbances, osteoporosis, myopathy, dyspepsia, myalgia and oedema	Biancone et al. (2008), Dalal (2007), Probert (2013), Witaicenis et al. (2012)
Biological agents	Infliximab, adalimumab and golizumab Vedolizumab, natalizumab etrolizumab Tofacitinib Mirikizumab and ustekinumab	TNF-α signaling inhibitors Integrin blocker JAK inhibitor IL12/IL13 antagonist	Delayed-type hypersensitivity reactions, itching, pain, neoplasia, congestive heart failure and tuberculosis	Hanžel and D’Haens (2020), Miehsler et al. (2010), Park and Jeen (2015), Targownik and Bernstein (2013), Vilar et al. (2007)
Immunosuppressive agents	Azathioprine Methotrexate Cyclosporine and tacrolimus	Protein synthesis DHFR inhibitor Calcineurin inhibitor	Hepatotoxicity, arthralgia, myalgia, leucopenia, bone marrow suppression, stomatitis, tremor, malaise, nephrotoxicity, neurological toxicity, gingival hyperplasia, and hirsutism	Bamba et al. (2011), Carter et al. (2004), Kawakami et al. (2015), Meier and Sturm (2011)
Surgical treatment	–	–	GIT disturbance, post-operative site-specific infections and psychological disadvantages	Meijs et al. (2014), Patel et al. (2013), Soon et al. (2014)

*COX* cyclooxygenase, *DHFR* dihydrofolate reductase, *IL* interleukin, *JAK* Janus kinase, *LOX* lipoxygenase, *MMP* matrix metalloproteinases, *NF-κB* nuclear factor kappa B, *PPAR-γ* peroxisome proliferator-activated receptor-γ, *TNF-α* tumor necrosis factor-α

## Drawbacks of conventional treatment approaches

The pharmacological therapies used for UC are associated with one or more side effects, which render them unsuitable for regular use. The conventional therapy is the treatment regimen, which is widely accepted and used by most of the healthcare professionals. The main adverse effects reported after using conventional therapy of UC include fever, nausea, headache, kidney damage, myopathy, myalgia, edema, neoplasia, congestive heart failure, tuberculosis, tremor, and hirsutism (Yokoyama et al. 2014). Side effects observed on using 5-aminosalicylates include bronchitis, arthralgia, headache, dizziness, abdominal cramps, and minor metabolic disorders (Patil and Moss 2008; Miehlke et al. 2014). Corticosteroids, though effective for UC when immediate remission is required, are also not free from side effects. The significant adverse effects of corticosteroids include edema, moon face, acne, mood disturbances, adrenal suppression, congenital fetal abnormalities, cushingoid face, gastric ulceration, and osteoporosis. Moreover, their long-term use may cause ocular side effects because of steroid-induced cataract and hyperglycemia, and chances of severe relapse have also been reported (Hanauer 2008; Kondamudi et al. 2013; Nunes et al. 2013). The safety profile of biologicals has also been studied. Their side effects include leukoencephalopathy, hypersensitivity, myalgia, neoplasia, congestive heart failure, tuberculosis, and malaise (Sands et al. 2014; Kondamudi et al. 2013; Langan et al. 2007). The most common side effects of immunosuppressants are hepatitis, pancreatitis, bone marrow toxicity, and leukopenia (Meier and Sturm 2011; Xu et al. 2004; Carter et al. 2004; Bamba et al. 2011)^.^

Surgical treatment (colectomy) in UC offers a better quality of life in life-threatening complications untreatable with medical therapy. Risk of colorectal cancer, sexual dysfunction, female infertility, reoccurrence of inflammation, and psychological disorders are associated with colectomy (Ingrid Ordás et al. 2012; Coviello and Stein 2014).

## Herbal approaches for the treatment of ulcerative colitis

**Fig. 1:**
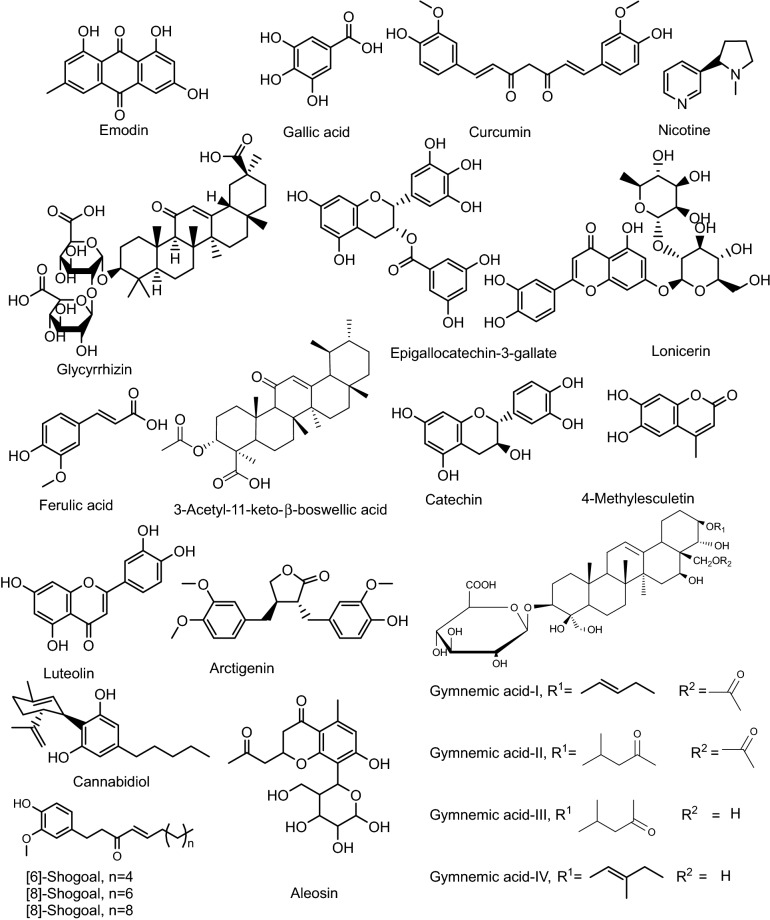
Chemical structures of bioactive constituents of herbal products posessing potential against UC

Herbal products are being used worldwide for their therapeutic potential in various ailments. The phytoconstituents such as catechins, flavonoids, terpenes, alkaloids, anthocyanins, quinines, and anthoxanthins having anti-inflammatory and antioxidant effects, can modulate the expression of pro-inflammatory signals and are considered potential agents for the treatment of UC (Zhang et al. 2006). All these agents act by multiple mechanisms, including suppression of TNF-α, IL-1β, cyclooxygenase (COX), lipoxygenase (LOX), and nuclear factor κB (NF-κB). Various bioactive principles of the plants, including gymnemic acid, shagol, catechin, curcumin, glycyrrhizin, boswellic acid, aloein, arctigenin, and cannabidiol, have been successfully employed to treat UC (Huang et al. 2010; Borrelli et al. 2009; Sałaga et al. 2014; Arun et al. 2014; Hsiang et al. 2013; Brückner et al. 2012). The chemical structures of some active constituents responsible for antiulcer activity have been represented in Fig. 1.

### Aloin

Aloin, the active principle of *Aloe vera* (AV) (Liliaceae), is known for its various biological activities, including hepatoprotective, antioxidant, anti-ulcer, anti-arrhythmic, antibacterial, antidiabetic and anti-ageing, anticancer, anti-inflammatory (Srinivas et al. 2013; Chandegara and Varshney 2013). Bioactive constituents present in aloe are anthraquinones (aloin, aloe-emodin, anthranol, and barbaloin), amino acids, hormones (auxin and gibberellins), steroids (cholesterol, campesterol, lupeol, and sitosterol) (Sahu et al. 2013; KB et al. 2014; Langmead et al. 2004).

**Fig. 2:**
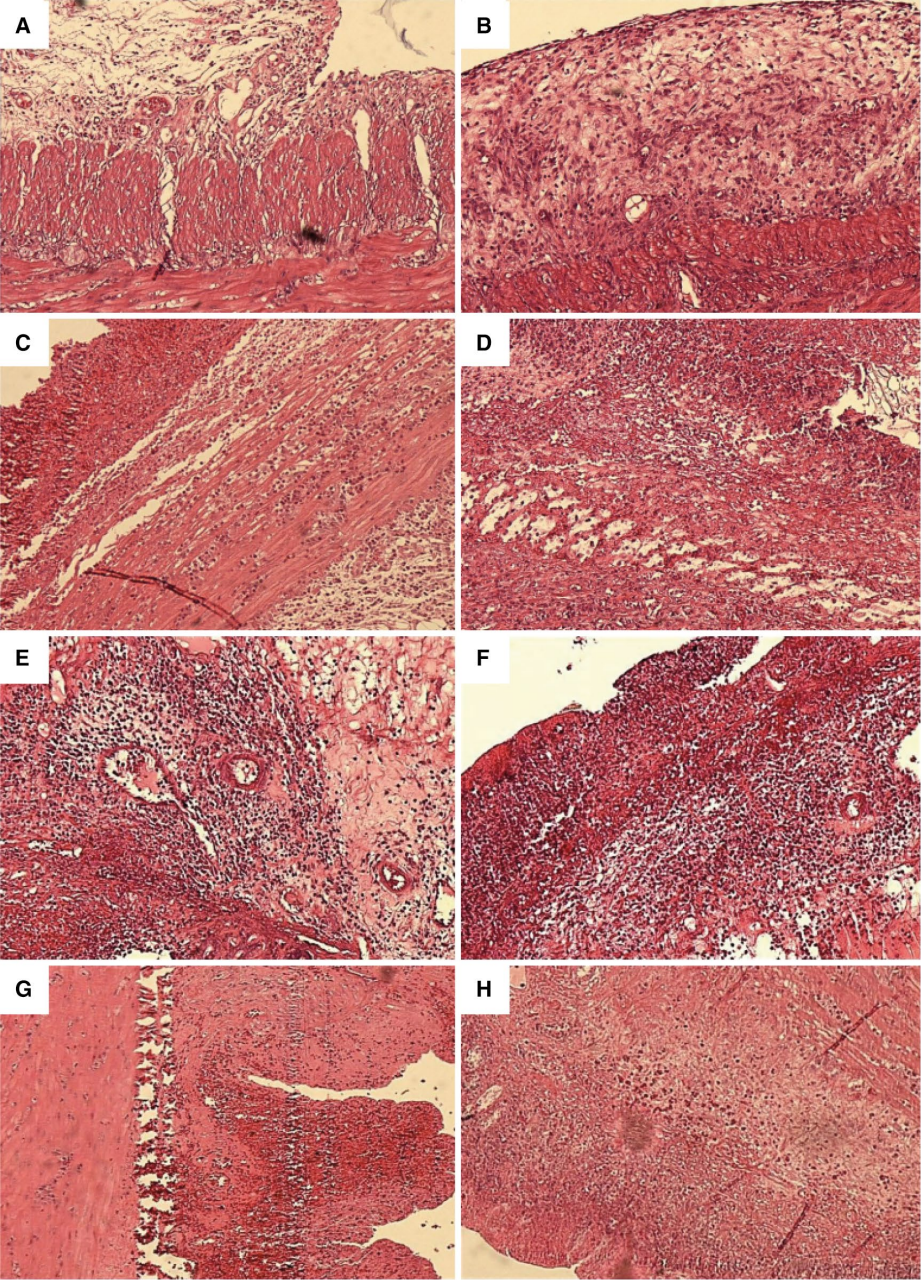
Photomicrographs of the rat colon stained with hematoxylin and eosin stain (× 40). Photomicrographs of protective **A** AV 50 mg/kg, **B** AV 300 mg/kg, **C** C+, **D** C−, and treatment groups, **E** AV 50 mg/kg, **F** AV 300 mg/kg, **G** C+, **H** C− in colitis rats. AV, *Aloe vera* (Bahrami et al. 2020)

**Fig. 3:**
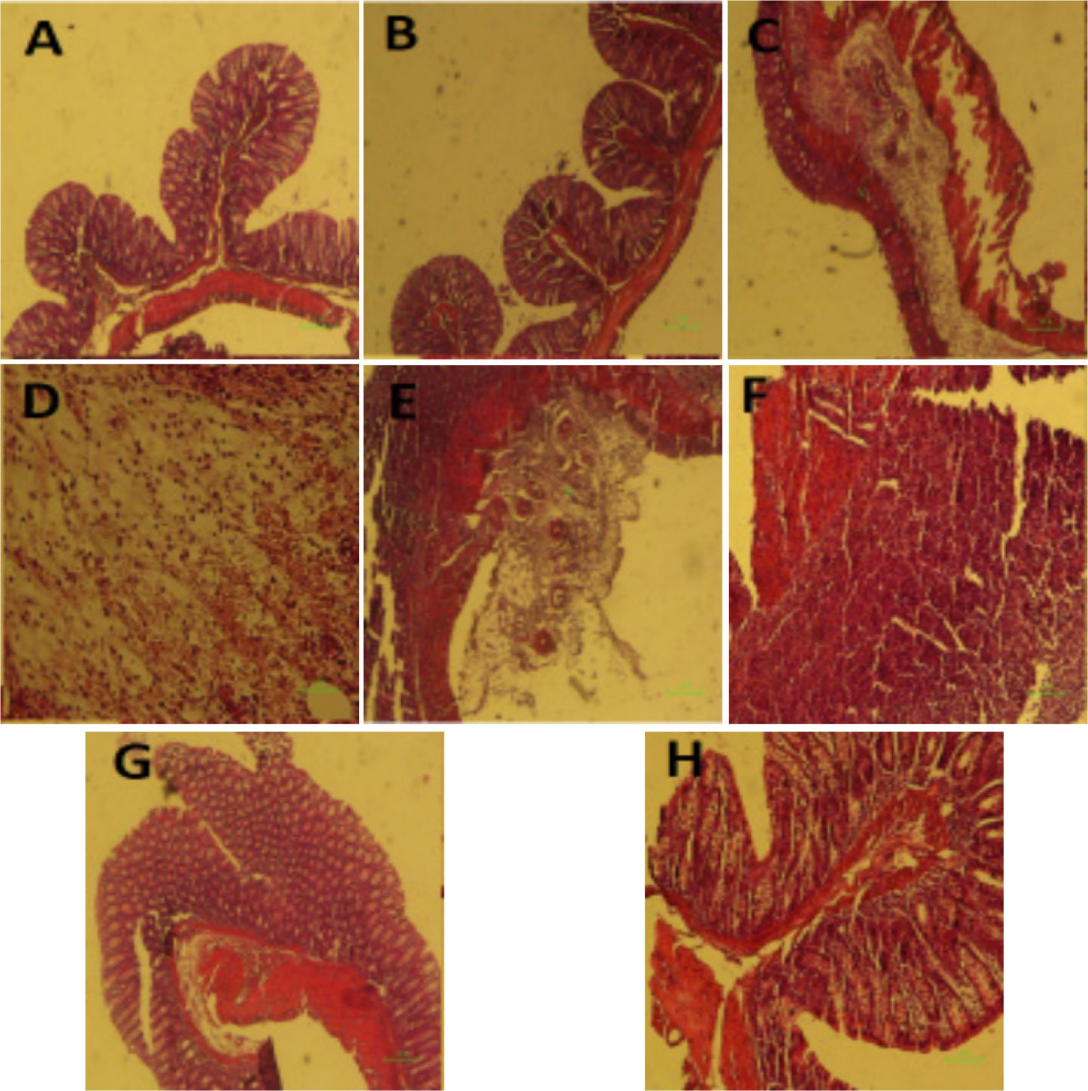
Representative of the microscopic slide sections of the colon samples in rats: **A** Normal colon sample magnification (× 40), showed the normal structure of mucosa with an intact epithelial surface, submucosa, and muscular layer. **B** Normal colon sample, magnification (× 100), showed the normal structure of layers. **C** Acetic acid-induced colitis with no treatment, magnification (× 40), showed an inflammatory reaction, presence of ulcer, inflammation, edema, and diffuse infiltration of leukocytes in the submucosal layer. **D** Acetic acid-induced colitis with no treatment, magnification (× 200), showed inflammation in the submucosal layer with ulceration and edema. **E** Acetic acid-induced colitis + oral administration of sulfasalazine, magnification (× 40), revealed inflammatory reaction in the serosa layer. **F** Acetic acid-induced colitis + oral administration of sulfasalazine sample, magnification (× 100) revealed inflammatory reaction with the dominance of lymphocyte and fewer neutrophils with tissue necrosis. **G**, **H** Acetic acid induced colitis + oral administration of aloe vera gel, magnification (× 40), and (× 100), revealed the improvement of inflammation and the normal tissue (Hassanshahi et al. 2020)

The role of aloe in the treatment of UC is mainly due to PGE2 and IL-8 secretion inhibition, which in turn, is responsible for its anti-inflammatory nature. It is further reported to inhibit ROS by phorbol 12-myristate 13-acetate (PMA) stimulated human neutrophils (Wan et al. 2014). Protective and therapeutic effects of AV gel on UC in acetic acid (AA)-induced colitis in rats have been evaluated by Bahrami et al*.* Reduction in inflammation, ulcer score, and tissue damage in AV-treated (50 and 300 mg/kg AV gel) rats compared with negative control animals (treated with 2 mL water), proved the usefulness in UC (Fig. 2). Pre-treatment with AV gel (50 and 300 mg/kg AV gel) reduced inflammation, lesions to serous layer and fibrosis and the results were found to be similar to positive control animals (treated with sulfasalazine 100 mg/kg) showed therapeutic effects in colitis animals (Bahrami et al. 2020). Hassanshahi et al. estimated the healing effect of AV gel in AA induced UC in rats. Histologically, it has been observed that AV gel treatment reduced and healed colon tissue damages in induced colitis. Also, this gel reduced apoptosis in rat’s colon, which showed a considerable decrease in Bax messenger ribonucleic acid (mRNA) expression and significantly increased B-cell lymphoma 2 (BCL-2) mRNA expressions. Further, the histopathological data have indicated protective effect of AV gel in colon, which was supported by reduced cell infiltration and appearance of normal tissue (Fig. 3) (Hassanshahi et al. 2020).

### Arctigenin

**Fig. 4:**
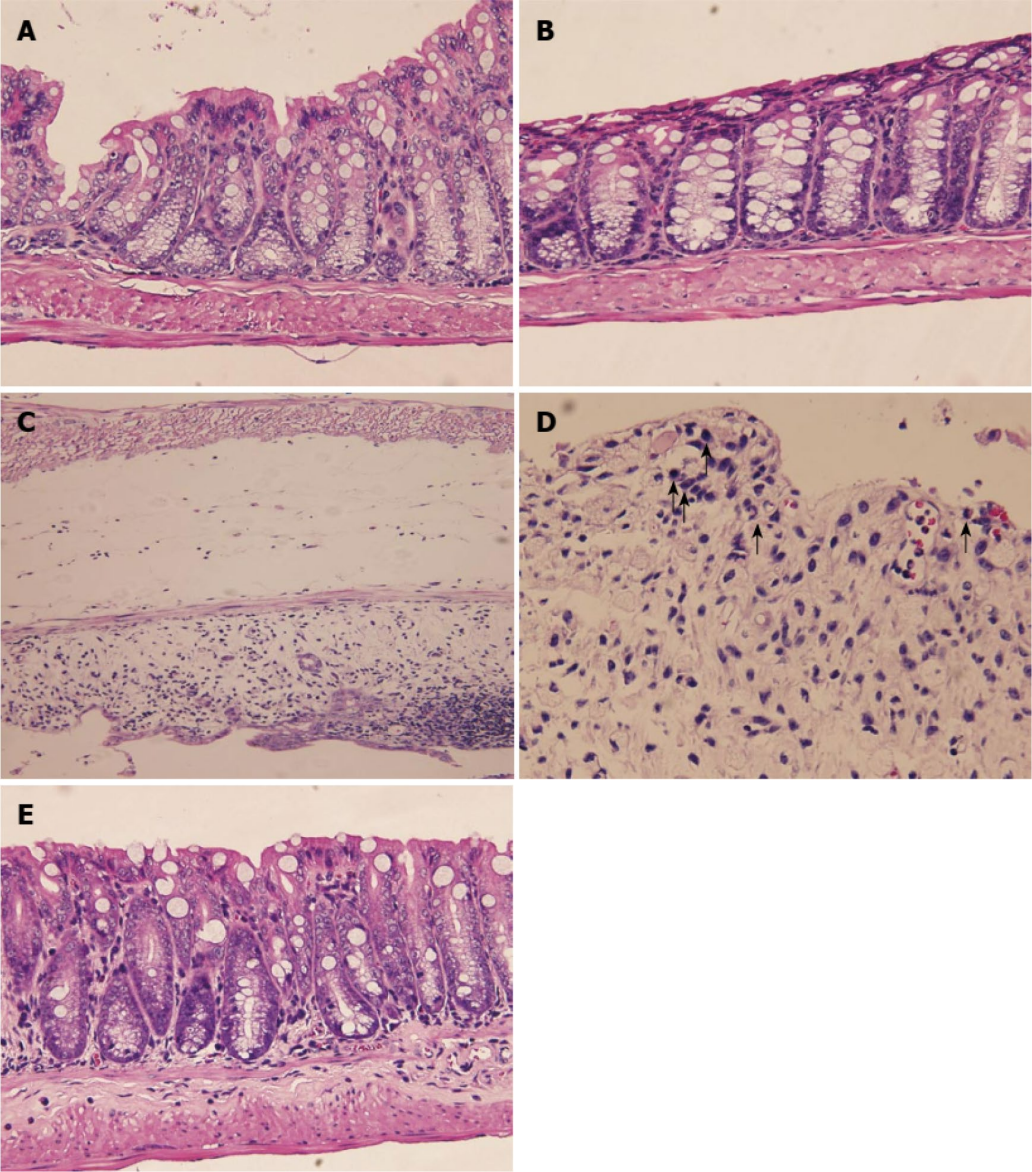
Histological analysis of mice. **A** Colon section from control (water treated) animals showing normal colon tissue architecture (HE, × 400); **B** Colon section from *Arctium lappa* L. (AL)-treated animals showing normal histological architecture with no inflammatory cell infiltration, edema or crypt abscesses (HE, × 400); **C**, **D** Colon section from dextran sulfate sodium (DSS)-treated animals showing severe submucosal erosion with edema, ulceration, inflammatory cell infiltration (indicated with arrows) and crypt abscesses as well as epithelioglandular hyperplasia (**C** HE, × 200, **D** HE, × 400); **E** Colon section from animals challenged with DSS after prior treatment with AL showing normal histological architecture with slight inflammatory cell infiltration and no submucosal edema or abnormality of crypt cells (HE, × 400). All the results are representative of three independent experiments (Huang et al. 2010)

*Arctium lappa* (AL) (Compositae), commonly known as Bardana or burdock, is widely used for various pharmacological activities such as diuretic, depurative, digestive, anti-inflammatory, antiulcer, antioxidant, antimicrobial, antirheumatic, and antiallergic (De Almeida et al. 2013; Zhao et al. 2014; Al-Snafi 2014; Kenny et al. 2014; Wang et al. 2014; Predes et al. 2011; El-Kott and Bin-Meferij 2015; Maghsoumi-Norouzabad et al. 2016; Liu et al. 2014). Its antiulcer activity is attributed to arctigenin and other secondary metabolites like dicaffeoylquinic acid, caffeoylquinic acids, chlorogenic acid, and caffeic acid (Chen et al. 2004; Jiang et al. 2016; Carlotto et al. 2015; Liu et al. 2012; de Almeida et al. 2012). Onopordopicrin, a secondary metabolite of AL, also has a protective effect on gastric mucosa and can be an effective remedy for UC. Huang et al*.* investigated the protective role of AL in a dextran sodium sulphate (DSS)-induced murine model of UC. The alteration in mean body weight and disease activity index (DAI) of diseased and AL-treated animals was found to be significant. Moreover, the histological findings showed that AL treatment could prevent mucosal edema, submucosal erosions, ulceration, inflammatory cell infiltration, and colon damage (Fig. 4). In case of control animals, the architecture of colon was found to be normal, whereas, pre-treatment with AL showed slight cell infiltration without any abnormality of crypt cells. Therefore, AL can be considered as effective in suppressing DSS-induced colitis and also for prevention of bloody diarrhea (Huang et al. 2010). The possible mechanism involved in protection is down regulation of inflammatory mediators like IL-6, TNF-α, macrophage inflammatory protein-(MIP)-2, monocyte chemo attractant protein (MCP)-1, mucosal vascular addressin cell adhesion molecule (MAdCAM)-1, intercellular adhesion molecule (ICAM)-1, T helper cell (Th) 1, Th17, inducible nitric oxide synthase (iNOS), mitogen-activated protein kinase (MAPK), and vascular cell adhesion protein (VCAM)-1 at both protein and mRNA levels in colonic tissues (Huang et al. 2010; Maghsoumi-Norouzabad et al. 2016).

Pomari et al*.* studied the effect of AL extract in treating UC. It has been found that AL elevates activities of antioxidant enzymes glutathione (GSH), superoxide dismutase (SOD), reduces lipid peroxidation (LPO), and prevents the formation of ROS; hence it can effectively be used for the treatment of UC (Pomari et al. 2014).

**Fig. 5:**
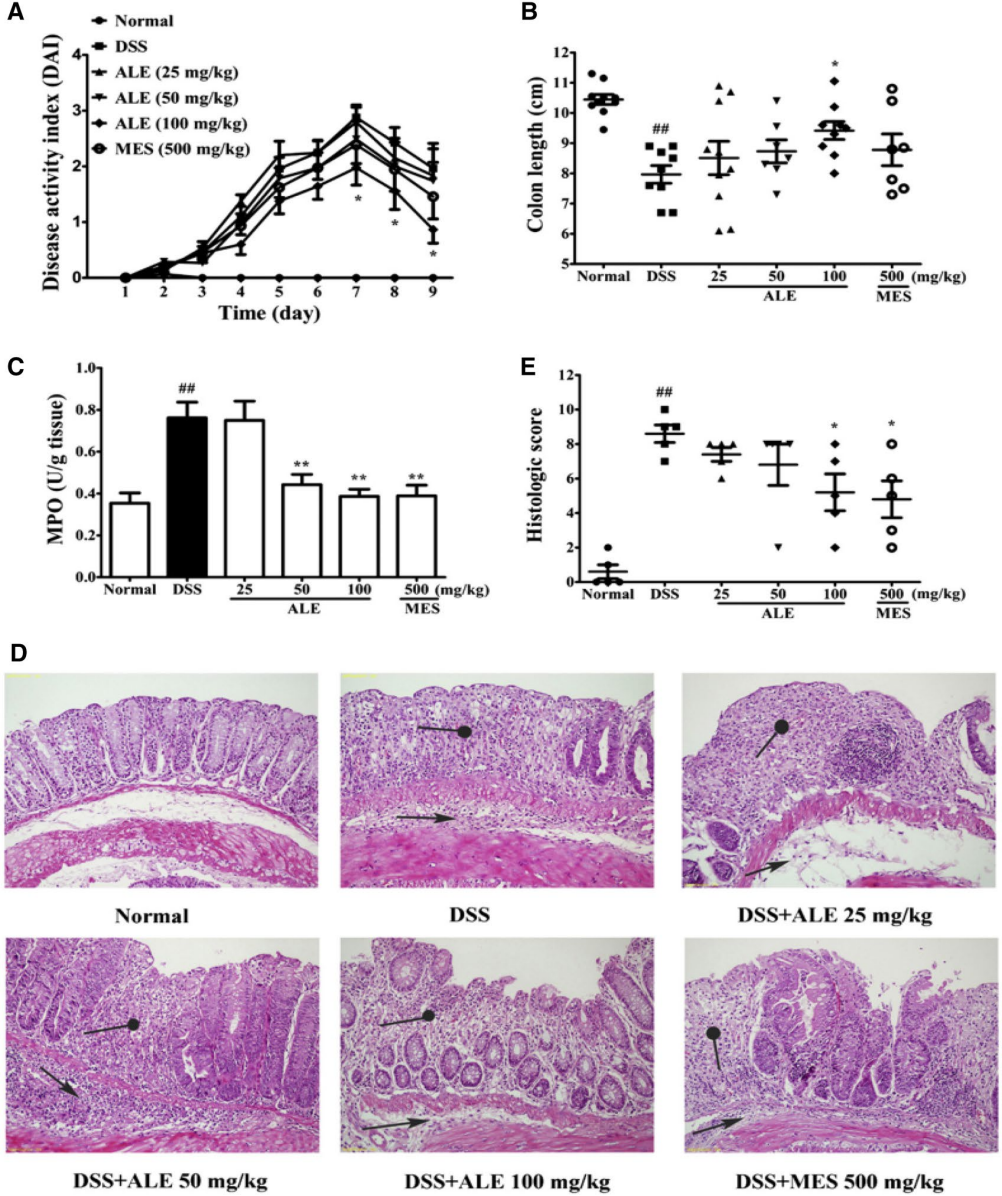
Effects of the ethanol extract of ALF on DSS-induced colitis in mice. Mice were treated with 3.5% DSS in drinking water for 7 days followed by drinking water for 3 days. ALE (25, 50, 100 mg/kg) were administrated orally once a day for 10 consecutive days. Mice were sacrificed at day 10. **A** DAI as the average score of body weight loss, stool consistency and rectal bleeding was scored from 0 to 4 (*n* = 7–8). **B** The colon length of each group at day 10 (*n* = 7–8). **C** The levels of MPO in colon (*n* = 7–8). **D** Histological changes of colon, characterized by distinct infiltration of inflammatory cells (arrow) and crypt destruction (solid circle) (magnification × 200). **E** Histological scores of colon from each group (*n* = 6). All data are presented as mean ± SEM. ^##^*P* < 0.01 vs. normal group, *P < 0.05, **P < 0.01 vs. DSS group. *ALE* ethanol extract of ALF, *MES* mesalazine (Wu et al. 2014)

Wu et al*.* evaluated the anti-colitis effect of arctigenin and arctiin in DSS-induced colitis in mice. The comparative studies indicated that reduction in weight loss, DAI, and histological damage in the colon were better observed with arctigenin. Furthermore, arctigenin recovered the loss of intestinal epithelial cells (E-cadherin-positive cells) and decreased the infiltration of neutrophils myeloperoxidase (MPO)-positive cells and macrophages (CD68-positive cells) and also caused down-regulation of TNF-α, IL-6, MIP-2, MCP-1, MAdCAM-1, ICAM-1, and VCAM-1. The above findings clearly indicate that arctigenin, not arctiin, is the active ingredient of AL for attenuating colitis (Fig. 5) (Wu et al. 2014).

### Boswellic acid

Boswellic acid (BA), obtained from *Boswellia serrata,* is a pentacyclic compound along with its various derivatives such as acetyl-11 keto-β-boswellic acid (AKBA), and 11-keto boswellic acid. The biological potential of BA and its various derivatives has been measured in the treatment of diseases like UC, asthma, bronchitis, laryngitis, cancer, inflammation, and pain (Iram et al. 2017; Anthoni et al. 2006; Ebrahimpour et al. 2017). In clinical evaluation, BA has been reported to reduce ulcer index, ulcer area in patients suffering from UC and was found to be well tolerated with minor gastrointestinal tract (GIT) disturbances (Algieri et al. 2015). Its anti-ulcer activity is attributed to the inhibition of pro-inflammatory enzymes such as COX-2, LOX-5, NF-ĸB, and leukotriene B4 (LTB4) (Ebrahimpour et al. 2017).

**Fig. 6:**
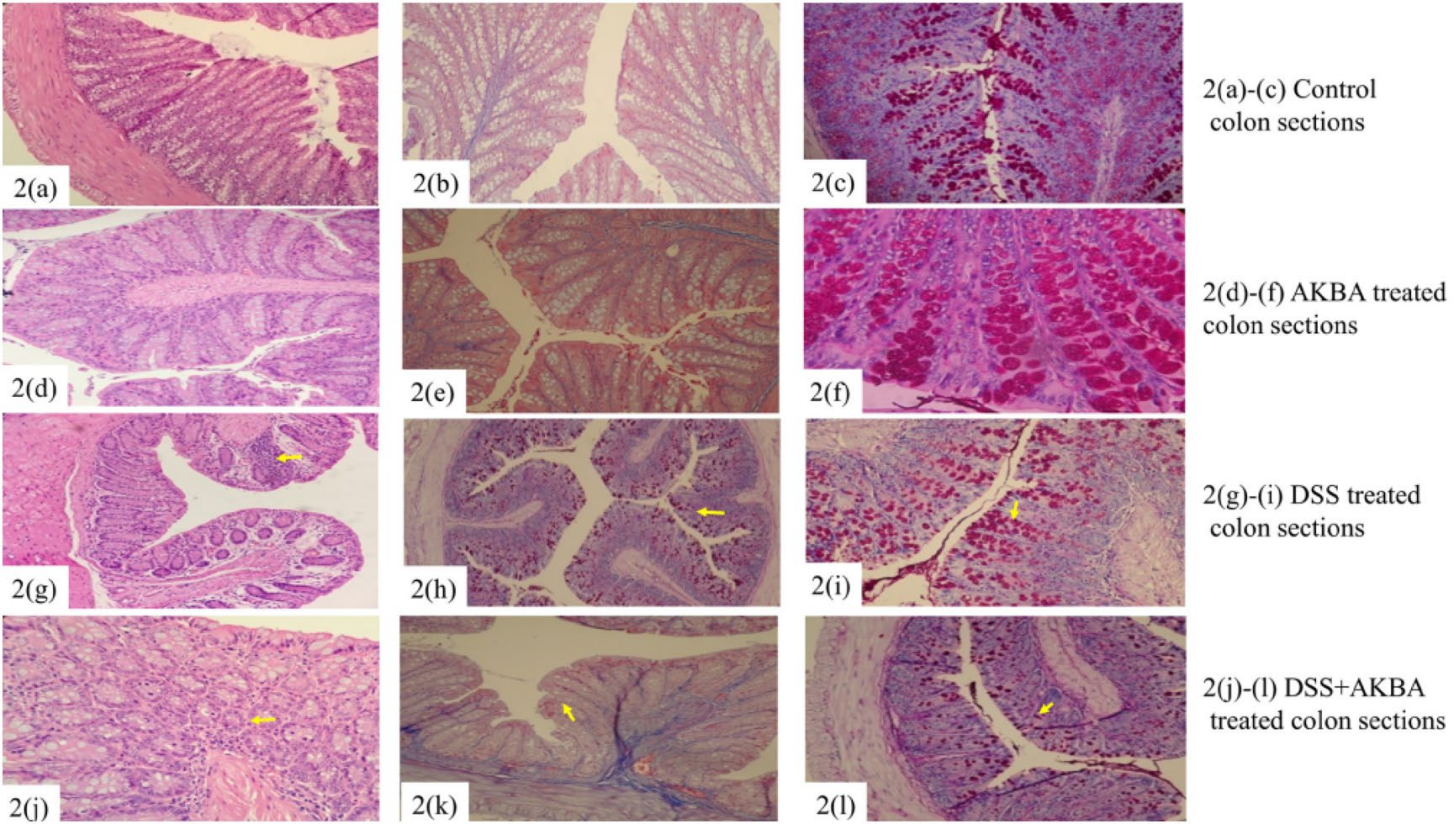
**a** Hematoxylin and eosin stain (H&E), **b** Masson’s trichrome (MT), and **c** Periodic acid-Schiff (PA) staining sections of control group colon with normal intact cross section structure. Panel **d** shows the AKBA treated tissue sections of colon H&E stained. Panel **e** colon section with MT stained. Panel **f** shows colon section PA stained with maintained histological structure. In Panel **g** the yellow arrow shows the infiltration of mixed leucocytes in cross section and H&E stained DSS treated colon. **h** DSS treated MT stained colon section with yellow arrow showing the infiltration of inflammatory cells. Panel **i** shows the DSS treated colon, PA stained specifying the mixed inflammatory cells infiltration sites in the tissue section. Panels **j**–**l** show the DSS + AKBA colon sections stained H&E, MT and PA. Yellow arrows represent the reduced infiltration of mixed inflammatory cells and well maintained tissue architecture (Roy et al. 2020)

Chande et al. reported that in patients with collagenous colitis, *B. serrata* extract was found to be effective in ameliorating disease process as compared to placebo (Chande et al. 2008). In another study, Catanzaro et al. evaluated the anti-inflammatory activity of *B. serrata* extract (BSE) and AKBA in colonic epithelial cell monolayers exposed to hydrogen peroxide (H_2_O_2_) or interferon (INF)-γ, TNF-α, an in vitro model of intestinal inflammation. Pre-treatment with BSE and AKBA significantly reduced functional and morphological alterations and the NF-κB phosphorylation induced by the inflammatory stimuli. Along with the same, BSE and AKBA also counteracted the increase of ROS caused by H_2_O_2_ exposure, therefore protecting the intestinal epithelial barrier from inflammatory damage and supported its use as a safe adjuvant for UC patients (Catanzaro et al. 2015). Roy et al*.* aimed to investigate the anti-inflammatory potential of AKBA against DSS-induced colitis in Swiss albino mice. Reduction in soreness and histopathological studies revealed that the chemo-protective effect of AKBA was attributed to anti-proliferation, apoptosis, and anti-inflammation (Fig. 6) (Roy et al. 2020).

### Catechin

*Camellia sinensis*, known as tea, is the most commonly consumed beverage globally. Tea is the primary source of many active constituents, including gallic acid, caffeine, epigallocatechin, catechins, and polyphenol, responsible for many health benefits like antiulcer, antioxidative, anticancer, anticarcinogenic, antiarteriosclerotic, hepatoprotective, and antimicrobial effects (Koo and Cho 2004; Roccaro et al. 2004; Olosunde et al. 2012; Pastore and Fratellone 2006; Lambert and Elias 2010; Ko et al. 2006; Osada et al. 2001; San Yeoh et al. 2016; Zanwar and Shende 2014; Donà et al. 2003; Hoensch and Oertel 2012; Lambert and Yang 2003; Yang et al. 2007; Ruhl and Everhart 2005; Hasegawa et al. 2011; Fernando and Soysa 2015).

Efficacy of Persimmon-derived tannin, i.e., condensed catechin, has been evaluated on a murine model of UC using DSS as ulcerogen by Kitabattake et al. The reduction in disease activity and inflammation through alteration of the microbiota composition and immune response established it as a promising candidate for UC therapy (Kitabatake et al. 2021).

Liu et al*.* explored the therapeutic potential of tea polyphenols in DSS-induced UC in mice, and the results indicated that they ameliorated intestinal inflammation and modulated gut microbiota (Liu et al. 2020).

### Curcumin

**Fig. 7:**
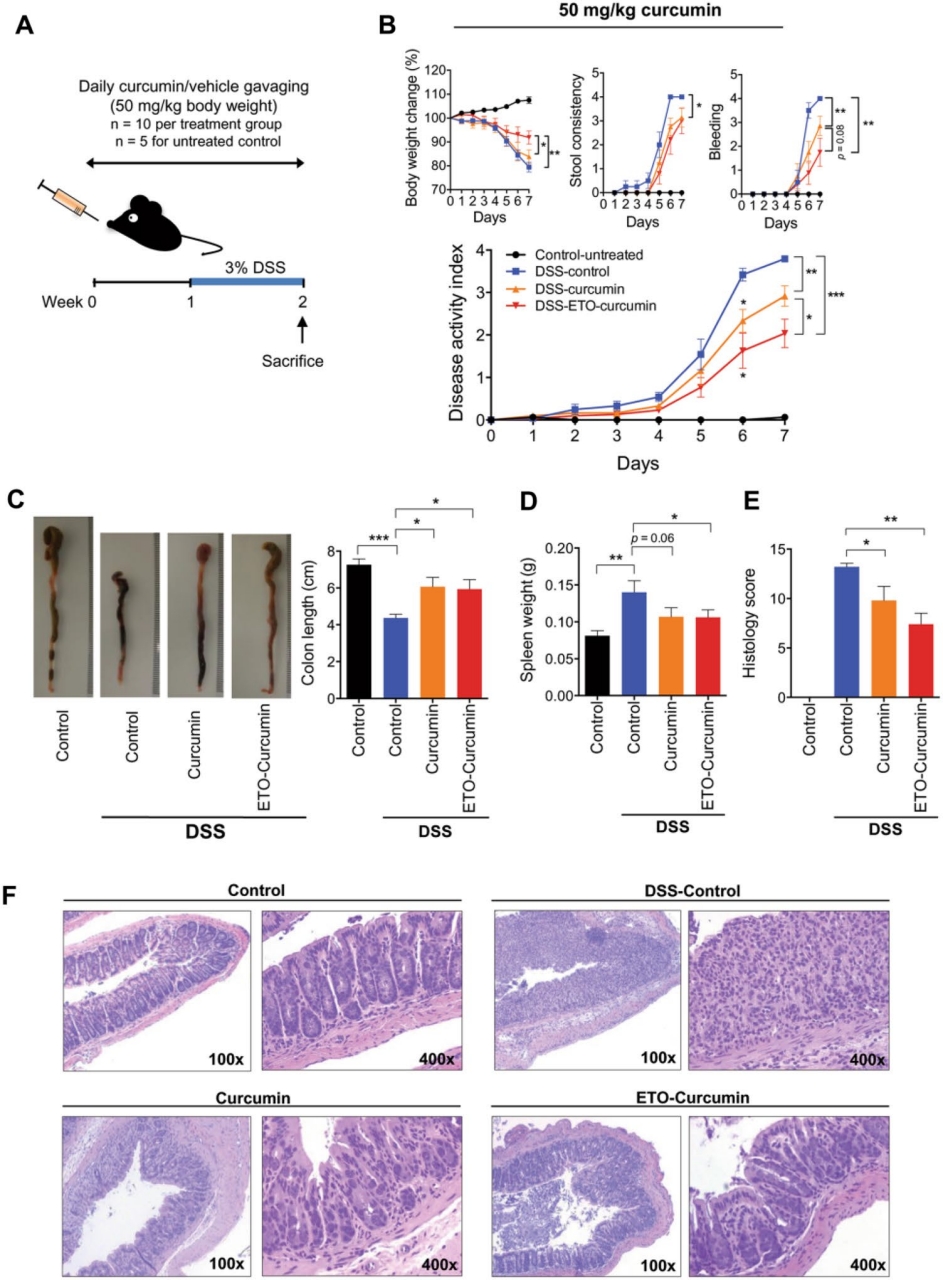
ETO-curcumin exerts superior anti-inflammatory effects compared to standard curcumin on DSS-induced inflammation at 50 mg/kg body weight. **A** Graphical representation of curcumin treatment strategy. **B** Changes in individual categories of disease activity index (DAI) (top), body weight changes, stool consistency and stool bleeding (left–right). Changes in DAI (bottom). **C** Representative image of colons (left) and average colon length (right). **D** Spleen weight and **E** histology score on day 14. **F** Representative hematoxylin and eosin (H&E) staining of large intestine on day 14. × 100 magnification (left) and × 400 magnification (right). *P < 0.05, **P < 0.01, ***P < 0.001(Toden et al. 2017)

Curcumin (diferuloylmethane) is a primary natural polyphenol found in the rhizome of *Curcuma longa* L. (Zingiberaceae). It is used to manage oxidative and inflammatory conditions, metabolic syndrome, arthritis, anxiety, and hyperlipidemia (Aggarwal and Harikumar 2009; Jurenka 2009; Anand et al. 2008). Chandan et al*.* investigated the efficacy of curcumin in ameliorating 2, 4, 6-trinitrobenzene sulfonic acid (TNBS)-induced colitis in mice. Results demonstrated the improvement in both wasting and histopathological signs in murine experimental colitis (Chandan et al. 2020; Sugimoto et al. 2002). Its postulated mechanism of action is suppression of NF-κB mediated IL-1β/TNF-α, which makes it an effective treatment option for inflammatory disorders (Sugimoto et al. 2002). Toden et al*.* investigated the anti-inflammatory activity of essential turmeric oils (ETO-curcumin) in an animal model of DSS-induced colitis against standard curcumin. ETO-curcumin improved DAI dose-dependently, while the anti-inflammatory efficacy of standard curcumin remained constant, suggesting that ETO-curcumin may provide superior anti-inflammatory efficacy compared to standard curcumin. The up-regulation in gene expression of anti-inflammatory cytokines in the colon, i.e*.*, IL-10, IL-11, and transcription factor of regulatory T-cells, i.e. Forkhead box P (FOXP)-3, further suggested that combined use of ETO and curcumin can afford better protection in UC (Fig. 7) (Toden et al. 2017).

### Glycyrrhizin

**Fig. 8:**
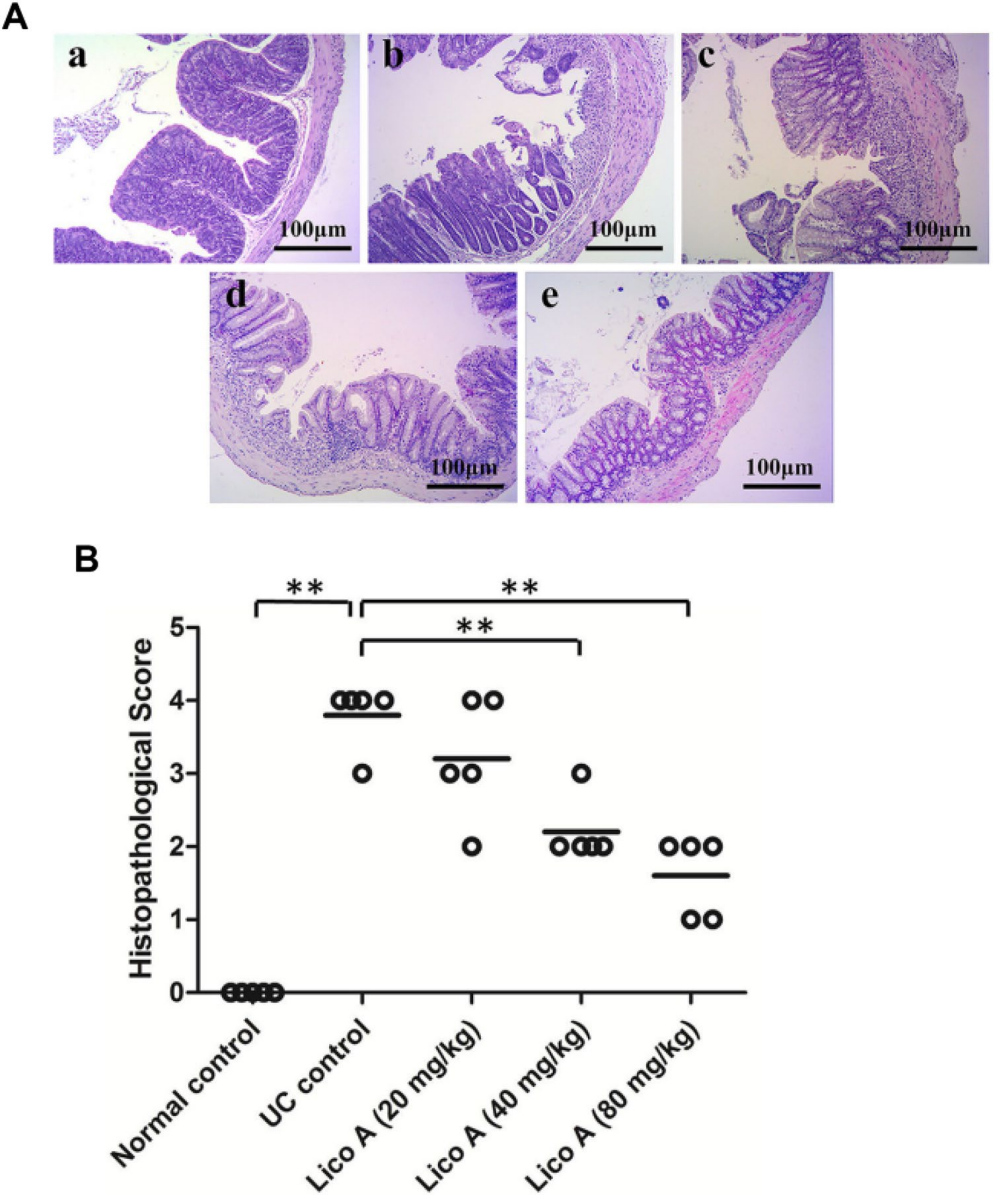
Treatment with Lico A relieved colonic microscopic damages in mice with DSS-induced UC. **A** Histological alteration of colonic mucosal. Histopathological sections were stained by H&E. Representative results are shown. Normal control (**a**); UC control (**b**) or Lico A, 20 mg/kg (**c**), 40 mg/kg (**d**) and 80 mg/kg (**e**). Original magnification × 200. **B** Histopathological scores of colons. Five specimens were selected randomly for histopathological study. Data are expressed as the means and individual histopathological scores, **P < 0.01 (Liu et al. 2018)

*Glycyrrhiza glabra* (Fabaceae), known as licorice, has been used to treat various ailments such as gastritis, bronchitis, ulcer, constipation, adrenal insufficiency, and allergy (Kim et al. 2006; Dogan and Ugulu 2013). Along with glycyrrhizin, it also contains other bioactive principles like glycyrrhizic acid, glycyrol, and sterol (Damle 2014). Liu et al*.* evaluated the protective effect of licorice flavonoids (LFs) in AA and DSS-induced colitis mouse model. Pre-treatment with LFs significantly reduced the wet weight/length ratio of the colon, percentage of the affected area, macroscopic and histological damage scores in both ulcer models. The LFs also decreased the oxidative stress and pro-inflammatory cytokines significantly, upregulated nuclear factor erythroid 2-related factor (Nrf)-2 pathway, and down regulated NF-κB pathway (Liu et al. 2017). Liu et al*.* investigated the anti-ulcerative activity of lichochalcone A (LicA) in DSS-induced UC in the mouse. Reduction in damage score, MPO, and colon length in a dose-dependent manner compared to the ulcer control group suggested its role as an anti-inflammatory agent. Further decrease in mediators of oxidative stress and inflammatory cytokines, down regulation of NF-ĸB, and up regulation of Nrf2 clarify its role in treating UC (Fig. 8) (Liu et al. 2018). Glycyrrhizin acts by inhibiting nitric oxide (NO), NF-ĸB, IL-6, IL-1β, TNF-α, and suppressing PGE_2_ level in lipopolysaccharide (LPS) stimulated macrophage (Kim et al. 2006; Dogan and Ugulu 2013).

### Gymnemic acid

**Fig. 9:**
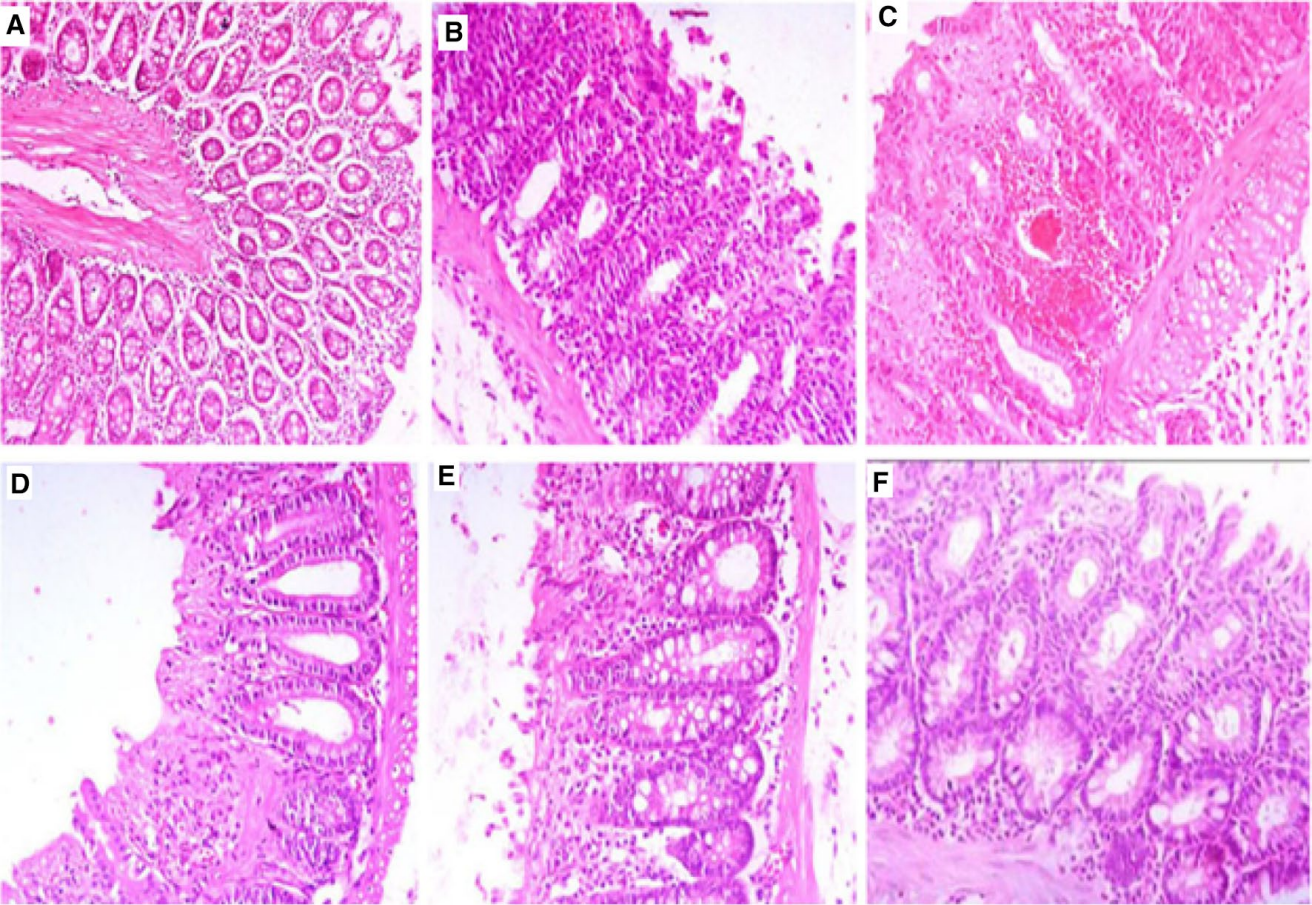
Histopathological sections of colons from rats stained with H&E (× 400). Colonic microscopic image of **A** normal rat colon from Cont group with intact mucosal layer and epithelial; **B** acetic acid (AA) treated rat colon with diffused active colitis, extensive damage including edema in submucosa and chronic inflammatory cells infiltrate with widely ulcerating mucosa, and hemorrhages; **C**–**E** dose dependent reparative epithelial changes and ulcer healing with lymphoid follicle in colon of GS treated rats (50, 100 and 200 mg/kg, respectively); **F** attenuated cell damage with complete ulcer healing in MES treated group (Aleisa et al. 2014)

It is obtained from *Gymnema sylvestre* (GS) (Asclepiadaceae), also known as Gurmur, which is native to India and also found in tropical forests of Africa, Australia, and Indonesia (Arun et al. 2014; David and Sudarsanam 2013; Gurav et al. 2007). Therapeutically, Gymnemic acid (GA) and its derivatives have been used to treat various diseases like diabetes, infection, inflammation, and oxidative stress (David and Sudarsanam 2013; Praveen et al. 2014; Ohmori et al. 2005; Jain and Devi 2016; El Shafey et al. 2013; Thakur et al. 2012). Rahman et al. have determined the free radical scavenging activity of GA by the 2,2-diphenyl-1-picryl-hydrazyl-hydrate (DPPH) model and suggested that it can be used to treat oxidative stress-related diseases (Rahman et al. 2014). Aleisa et al*.* evaluated the potential of GS leaves\ extract in AA induced UC in Wistar rats against a standard drug, mesalazine. Pretreatment with GS showed the inhibition of thiobarbituric acid reactive species (TBARS) elevation and mucus content; GSH reduction and enzymatic level of SOD and catalase (CAT) were brought to normal in a dose-dependent manner. The histopathological screening indicated dose-dependent reparative epithelium changes in the colon of GS-treated animals. Further, GS exhibited reparative epithelial damage and healing of lymphoid follicle (Fig. 9). The anti-ulcerative activity of GS was attributed to inhibition of TNF-α, SOD, CAT, GSH, IL-1β, IL-6, PGE, and NO (Aleisa et al. 2014).

### Lonicerin

**Fig. 10:**
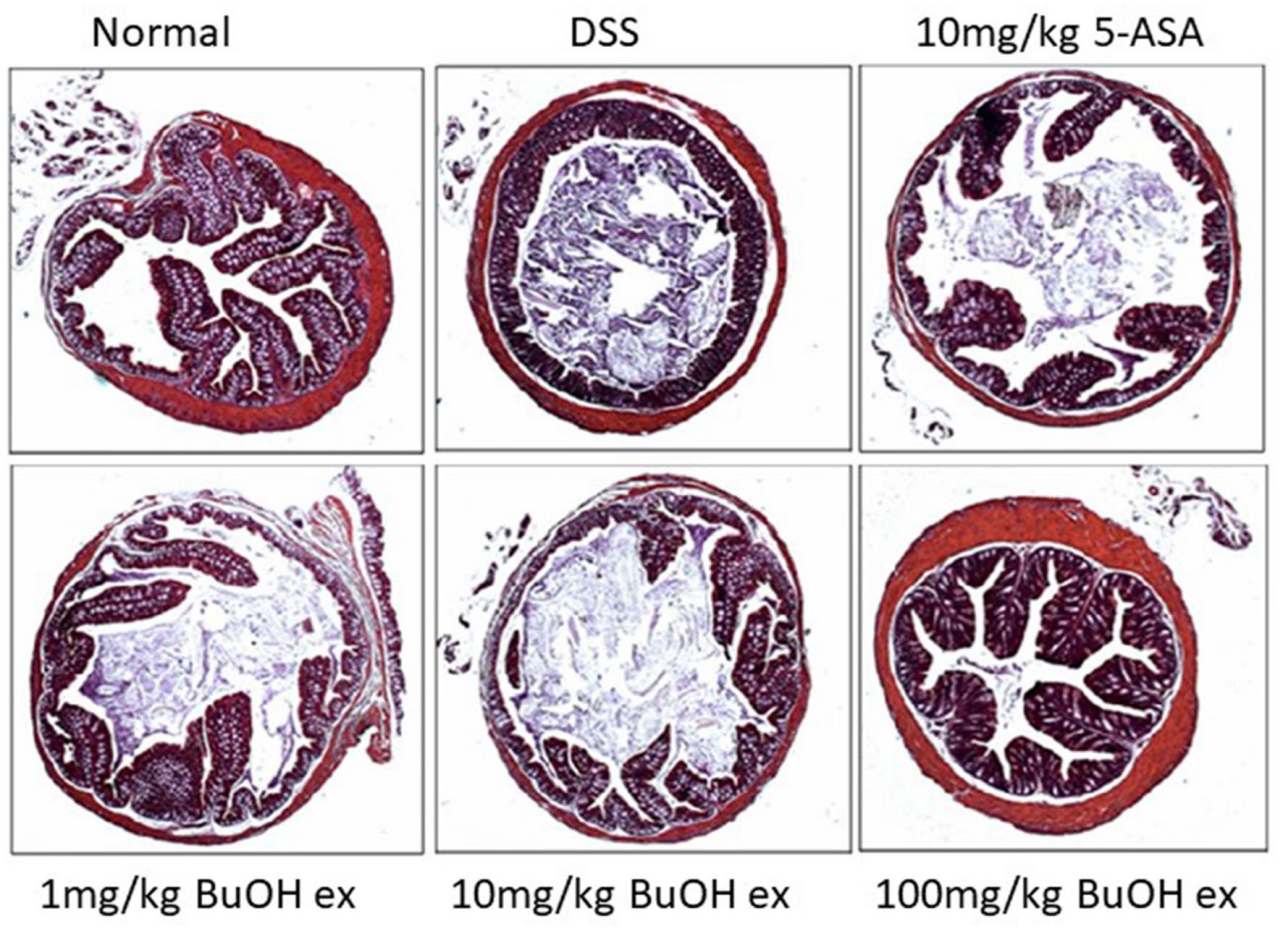
Microscopic study (original magnification × 50) of colons of mice with DSS-induced colitis treated with BuOH extracts of *L. japonica*. Treatment dose was 1 mg/kg, 10 mg/kg and 100 mg/kg, respectively, and 5-ASA was 100 mg/kg (Lee et al. 2011)

**Fig. 11:**
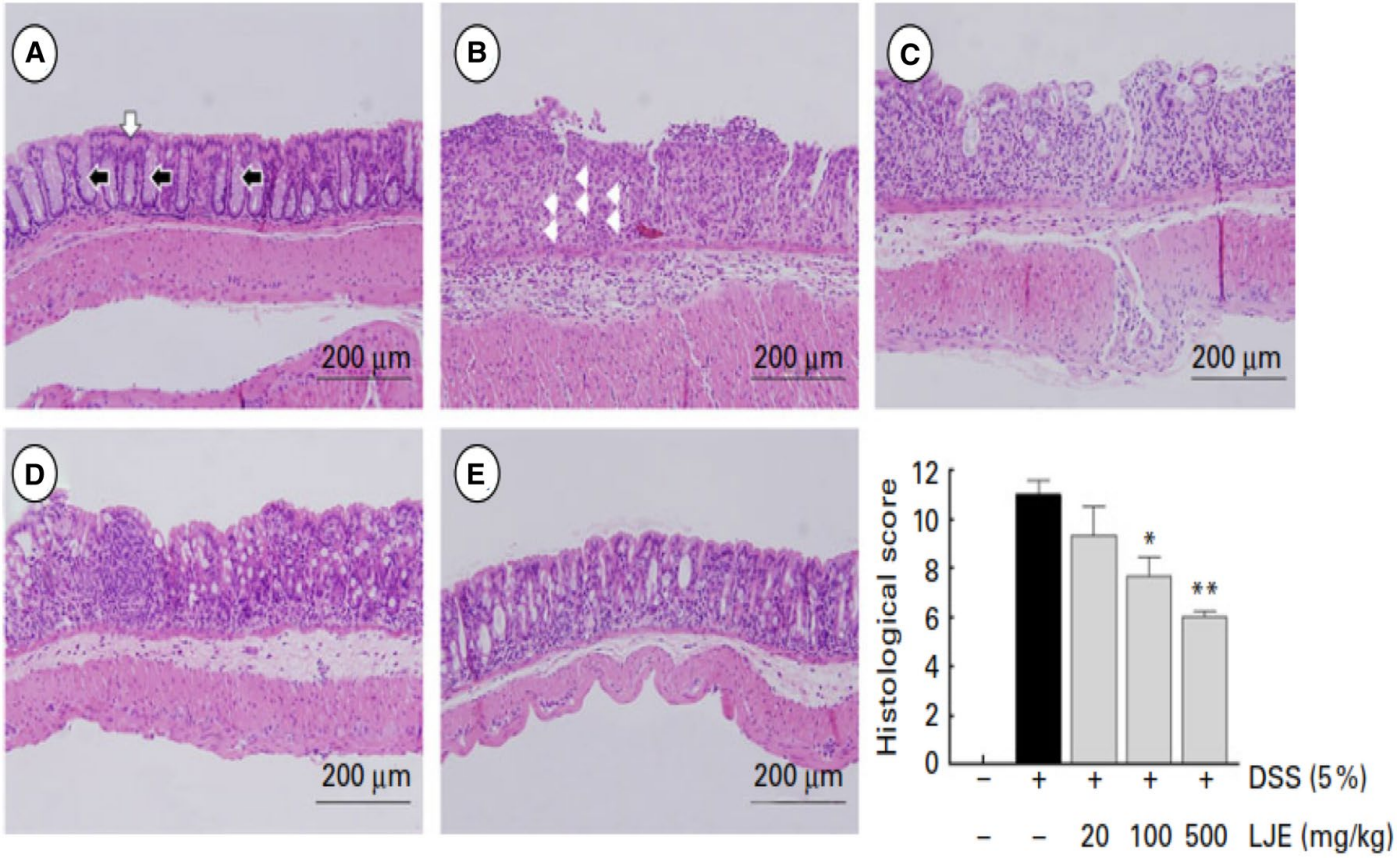
Effects of the water extract of *Lonicera japonica* (LJE) on histological findings and score in dextran sulphate sodium (DSS)-induced colitis. The images **a**–**e** are the representative histological findings of all groups: **a** normal, **b** DSS, **c** DSS þ LJE 20 mg/kg, **d** DSS þ LJE 100 mg/kg and **e** DSS þ LJE 500 mg/kg groups, cryptal grand, surface of the epithelium;, neutrophils. The graph shows the histological scores. The score of the normal group is zero. Values are means (*n* = 8 for each group), with their standard errors represented by vertical bars. Mean values were significantly different compared with the DSS group: *P < 0·05, **P < 0·01 (Park et al. 2013)

Lonicerin obtained from *Lonicera japonica* Thunb. known as *Japanese honeysuckle* has been used as antibacterial, anti-inflammatory, antiviral, antiendotoxin, blood fat reducing, and antipyretic (Shang et al. 2011). The protective role of a new polysaccharide isolated from *L. japonica* Thunb. (LPJ) against DSS-induced UC has been estimated in mice by Zhou et al. Further, its effects on intestinal flora and immune response were also studied. Significant increase in body weight, serum cytokines parameters (IL, TNF-α, and IFN-γ), secretory immunoglobulin A (SIgA) concentration, and natural killer (NK) cells and cytotoxic lymphocyte (CTL) activities were observed in DSS-treated mice. Improvement in the number of intestinal probiotics (*Bifidobacterium* and *Lactobacilli*) and decrease in number of the pathogenic bacteria (*Escherichia coli* and *Enterococcus*) has been observed with LJP-treated rats in a dose-dependent manner (Zhou et al. 2021). Lee et al*.* explored the effect of butanol extract of *L. japonica* in reducing the DSS-induced colitis and crypt injury in mice. The effectiveness of *L. japonica* in alleviating colitis was observed in dose dependent manner and was also found to be comparable with standard control i.e. 5-amino salicylic acid (5-ASA) as no distortion of crypt and cell infiltration was observed in treatment control group animals (Fig. 10) (Lee et al. 2011). Park et al. investigated the prophylactic effects of LJP on DSS-induced colitis in BALB/c mice. The LJP caused inhibitory effects against colon shortening, weight loss, and histological damage in a dose-dependent manner. The extract of *L. japonica* (LJE) also down-regulated IL-1b, TNF-α, INF-γ, IL-6, IL-12, and IL-17. The down-regulation of histological score was observed in dose dependent manner in LJE treated animals. The protective effect of LJE against histological damage of colonic mucosal layer as relatively intact epithelium was observed as compared to DSS treated animals (Fig. 11) (Park et al. 2013). Lv et al*.* verified the potency of lonicerin in UC as it disrupts the NLRP3–ASC–pro-caspase-1 complex assembly dose-dependently and therefore alleviates colitis. Therefore, lonicerin can be considered as a potent anti-inflammatory epigenetic agent and a novel approach to treat UC (Lv et al. 2021).

### Shagoal

Ginger, *Zingiber officinale* Roscoe (Zingiberaceae), is a natural dietary rhizome with various biological properties and activities. The health benefits of ginger are attributed to numerous biological components, including gingerols, gingerdiols, shogaols, paradols, and zingerones. Further, shogaol, a primary active ingredient of ginger, exists in various forms such as 4-, 6-, 8-, 10-, and 12-shogaol (Gupta et al. 2021).

Guo et al*.* (2021) investigated the therapeutic action of ginger in DSS-induced UC in male BALB/c mice and reported that ginger alleviated colitis-associated pathological changes and decreased the mRNA expression levels of IL-6 and iNOS.

**Fig. 12:**
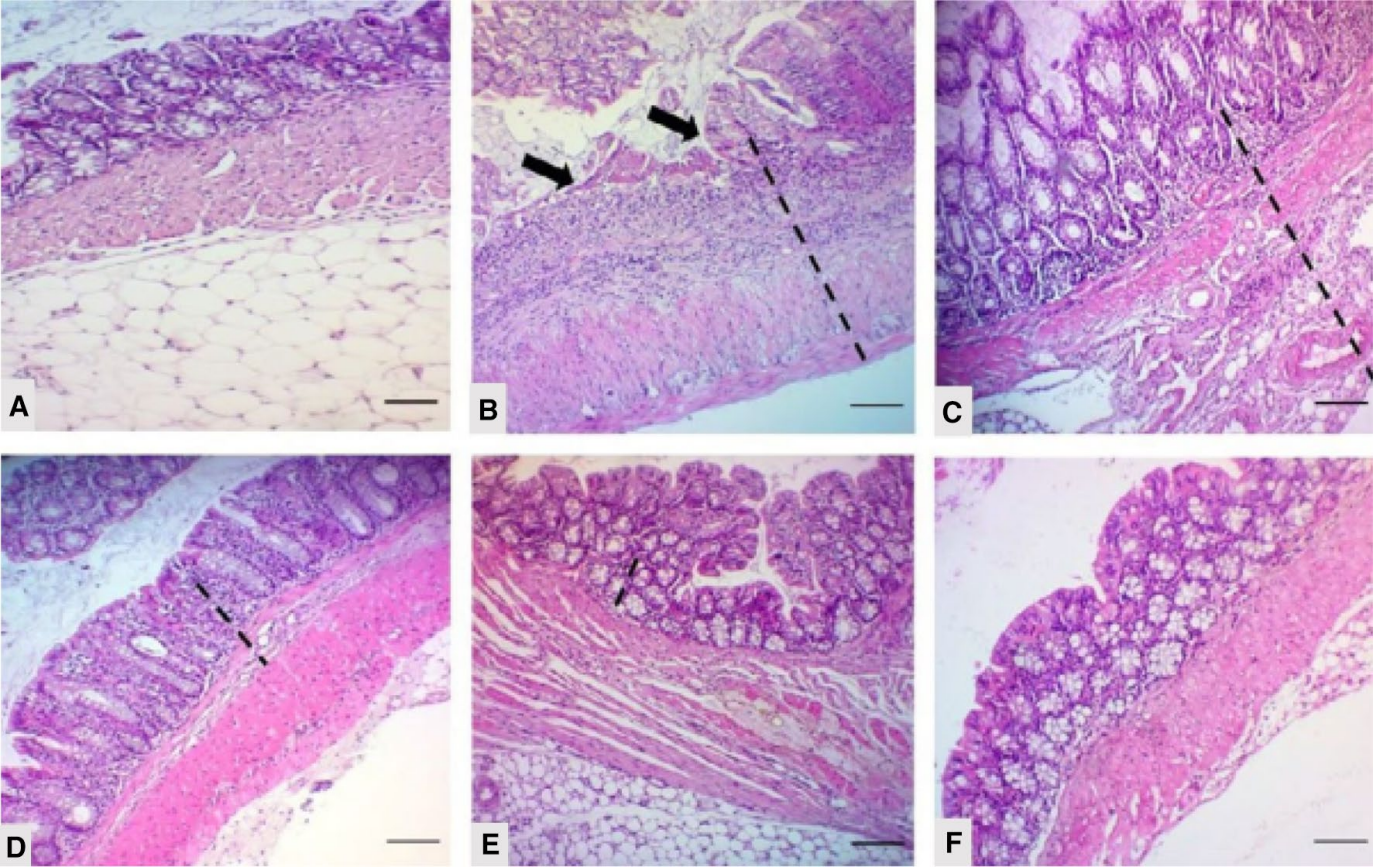
Microscopic view and the total histological index score of the proximal colon of mice in all groups of the current study. **a** Group 1 (negative control): intact epithelium with normal epithelial cells infiltration (Sum score 0); **b** Group 2 (control + ve DSS exposure without treatment): focal epithelial ulceration (black arrows) with transmural infiltration of inflammatory cells (Sum score 5); **c** Group 3 (vehicle control group): intact epithelial surface with transmural infiltration of inflammatory cells (Sum score 4); **d** Group 4 (DSS exposure and 6-TG treatment): intact epithelial surface with moderate infiltration of inflammatory cells in mucosa and submucosa (Sum score 2); **e** Group 5 (DSS exposure and 20 mg/kg BW Shogaol treatment): intact epithelium with mild infiltration of inflammatory cells in mucosa only (Sum score 1); **f** Group 6 (DSS exposure and 40 mg/kg BW Shogaol treatment): intact epithelium with no inflammatory cells infiltration (Sum score 0). H&E stain; Black dash line indicated the extent of inflammatory cells infiltration; scale bar 100 μm. *DSS* dextran sodium sulfate, *BW* body weight, *6-TG* 6-thioguanine (Hassan and Hassan 2018)

**Fig. 13:**
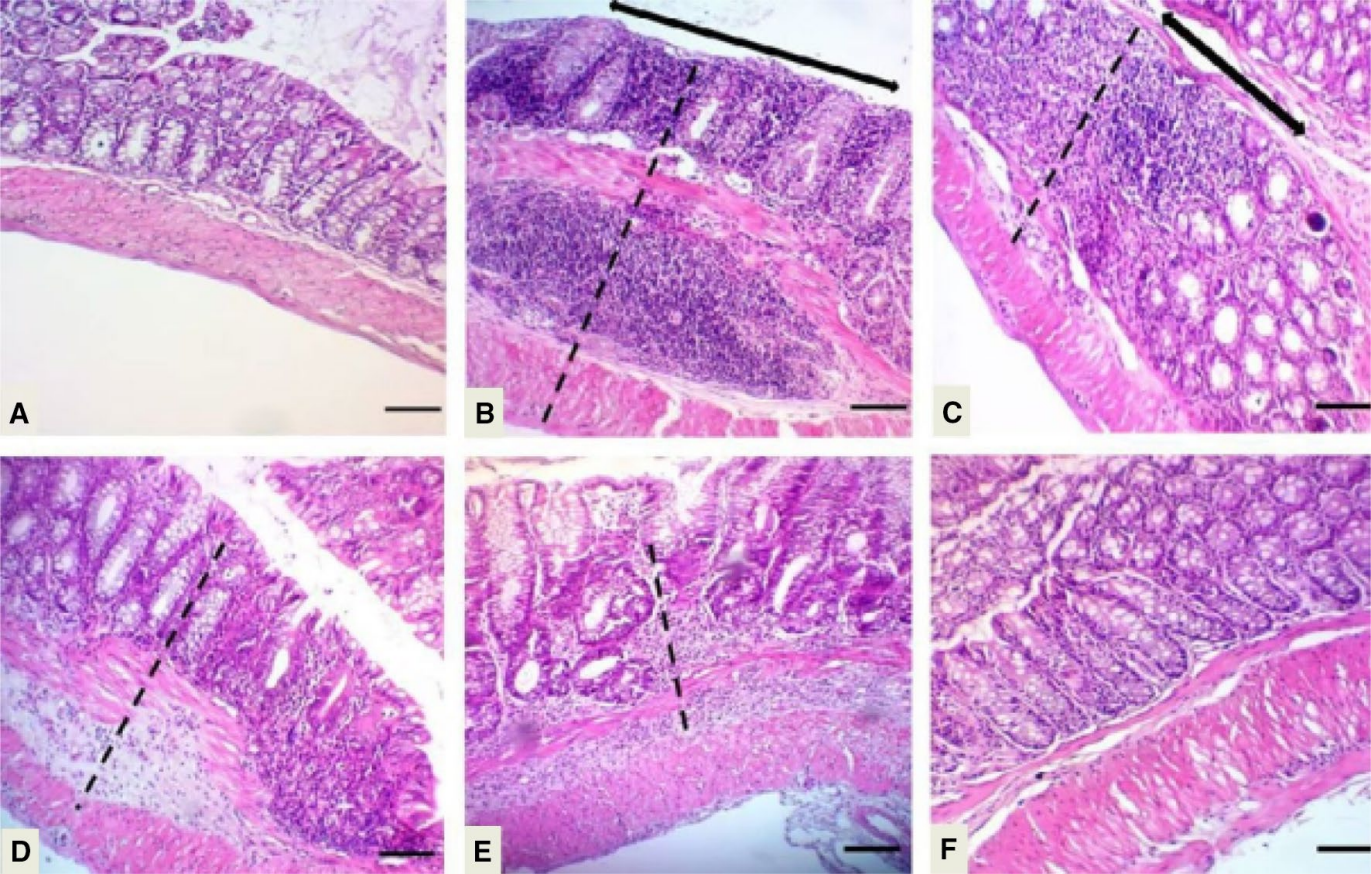
Microscopic view and the total histological index score of the distal colon of mice in all groups of the current study. **a** Group 1 (negative control): intact epithelium with normal epithelial cells infiltration (Sum score 0); **b** Group 2 (control + ve DSS exposure without treatment): extensive epithelial ulceration (black arrows) with transmural infiltration of inflammatory cells (Sum score 6); **c** Group 3 (vehicle control group): focal epithelial erosion (black arrow) with transmural infiltration of inflammatory cells (Sum score 4); **d** Group 4 (DSS exposure and 6-TG treatment): intact epithelial surface with moderate infiltration of inflammatory cells in mucosa and submucosa (Sum score 2); **e** Group 5 (DSS exposure and 20 mg/kg BW Shogaol treatment): intact epithelium with moderate infiltration of inflammatory cells in mucosa and submucosa (Sum score 2); **f** Group 6 (DSS exposure and 40 mg/kg BW Shogaol treatment): intact epithelium with mild infiltration of inflammatory cells in the mucosa (Sum score 1). H&E stain; Black dash line indicated the extent of inflammatory cells infiltration; scale bar 100 μm. *DSS* dextran sodium sulfate, *BW* body weight, *6-TG* 6-thioguanine (Hassan and Hassan 2018)

Zhang et al*.* (2017) explored the potency of orally administered siRNA-CD98/ginger-derived lipid vesicles (GDLVs) targeting specifically the colon tissues, which resulted in reduced expression of CD98 in colitis, thereby suggesting the use of these nanovesicles for UC. Hassan and Hassan explored the effect of shogoal in DSS-induced UC in BALB/c mice and compared their effect to that of an immunosuppressant drug, 6-thioguanine. The reduction in DAI and the histopathological score of shogoal treated rats demonstrated its beneficial role in treating UC. In Figs. 12 and 13, the efficacy of different concentration of shogoal was shown in proximal and distal parts of colon, respectively. The positive control group (DSS-exposed animals without treatment) showed focal epithelial ulceration with transmural infiltration of inflammatory cells whereas intact epithelial surface with normal epithelial cells infiltration was observed in negative control group animals. Shogoal showed protective effect in dose dependent manner with mild infiltration of inflammatory cells. Further, the histological index score of the proximal colon of mice was found to be maximum i.e. 5 for DSS-exposed animals without treatment and 0 for DSS-exposed shogoal treated (40 mg/kg BW) animals (Fig. 12). However, the histological index score of the distal colon of mice was found to be maximum 6 and minimum 1 for DSS-exposed animals without treatment and DSS-exposed shogoal treated (40 mg/kg BW) animals, respectively (Fig. 13) (Hassan and Hassan 2018). It has been further documented that the antiulcerogenic activity of shagoal is due to the suppression of NF-kβ, TNF-α, and IL-1 β signalling pathway (Hsiang et al. 2013; Banerjee et al. 2011).

### Miscellaneous phytoconstituents

Tannins obtained from rhatany root (*Krameria triandra*), wine grape seed (*Vitis vinifera*), and Scotch pine bark (*Pinus sylvestris*) have also been used effectively for UC. Their efficacy is directly related to the presence of proanthocyanidin. Higher the proanthocyanidin content; more is the ability to combat inflammation by inhibiting NF-κB p65 activity, decreased matrix metalloproteinase (MMP) production responsible for damage on GI mucosa (Clinton 2009). Cinnamon oil also has a role in preventing colonic damage in a dose-dependent manner and has a considerable effect on body weight gain recovery (Bujňáková et al. 2013).

Bruckner et al. studied the effect of polyphenol epigallocatechin-3-gallate (EGCG) of tea in DSS-induced colitis mice. The reduced level of malondialdehyde (MDA) and MPO as well as enhanced expression of SOD, glutathione peroxidase (GPO), and pro-inflammatory cytokines have depicted its potential to treat UC (Brückner et al. 2012).

Liu et al*.* described the protective effect of a polysaccharide from *Rheum tanguticum* as an antiulcerogenic agent in TNBS-induced UC in rats. Significant inhibition of NF-Κβ, Th1/Th2 cytokine production was observed in a dose-dependent manner (Liu et al. 2003, 2005, 2008, 2009).

The safety and efficacy of ethanolic extract of *Scorzonera alexandrina* were evaluated in Wistar albino rats with AA-induced colitis. The findings demonstrated a significant reduction in inflammation and acute colonic damage due to the presence of luteolin and luteolin 7-O-glycoside. The results were associated with the ROS scavenging property of the plant (Akkol et al. 2012; Donia 2016).

Witaicenis et al*.* (2012) explored the anti-inflammatory activity of 4-methylesculetin, a natural coumarin, obtained from *Scopolia carniolica*, in TNBS-induced colitis rat and a significant decline in the level of reduced IL-1β, TNF-α, and oxidative stress, confirmed its role as antiulcer agent. *Typha angustifolia* has been tested for its anti-inflammatory activity, and results have shown attenuation in GSH depletion and decrease in MPO, and alkaline phosphate (AP) activity because of which it can be used in UC (Fruet et al. 2012; Chen et al. 2017). The possible mechanism of AP involved in curing colitis may be attributed to its dephosphorylation of pro-inflammatory molecules such as LPS, flagellin and adenosine triphosphate, which are released from cells under stressed conditions during inflammation (Lukas et al. 2010; Bilski et al. 2017).

The role of cannabidiol (CBD), a non-psychotic component of *Cannabis sativa*, was investigated in a murine model of dinitrobenzene sulfonic acid (DNBS)-induced colitis in rats by Borrelli et al., and the effect of CBD in the change of body weight and colon weight/colon length ratio was determined. Results indicated that treatment with CBD (1–10 mg/kg) significantly reduced the colonic damage associated with DNBS administration. No significant change in COX-2 expression was observed; however, over-expression of iNOS, nitrile production, IL-1β, and IL-10 was found to be declined up to a considerable extent (Borrelli et al. 2009).

Kotakadi et al*.* estimated the effect of *Ginkgo biloba* extract (EGB) in amelioration of inflammatory injury in TNBS-induced colitis in rats with different doses. The inflammatory response was assessed by histology and measurement of MPO, GSH, TNF-α, and IL-1β levels in the colon mucosa. It significantly decreased the colonic MPO activity, TNF-α, and IL-1β levels. The increased GSH concentration was observed; hence it can be used to treat UC due to its scavenging activity (Kotakadi et al. 2008). The antiulcer activity of many herbal products has been summarized in Table 2.

**Table 2:** Phytoconstituents and their molecular targets

Phytoconstituent	Chemical class	Source	Family	Target	References
Aloin	Anthraquinones	*Aloe vera*	Liliaceae	IL-8, PGE_2_, SOD, ROS	Wan et al. (2014)
Apigenin	Flavonoid	*Scorzonera alexandrina*	Asteraceae	ROS, PGE_2_	Akkol et al. (2012)
Arctigenin	Lignan	*Arctium lappa*	Compositae	IL-6, TNF-α, MIP-2, MCP-1	Al-Snafi (2014), Huang et al. (2010)
Boswellic acid	Pentacyclic terpene	*Boswellia serrata*	Burseraceae	COX-2, LOX-5, NF-ĸB, LTB_4_	Ebrahimpour et al. (2017), Iram et al. (2017), Roy et al. (2020)
Cannabidiol	Chromone	*Cannabis sativa*	Cannabaceae	IL-1β, IL-10, NO, COX-2	Borrelli et al. (2009)
Catechin	Polyphenol, flavanoid	*Camellia sinensis*	Theaceae	TNF-α, IL-6, IL-γ, SOD	Hasegawa et al. (2011), San Yeoh et al. (2016)
Curcumin	Diferuloylmethane	*Curcuma longa*	Zingiberaceae	MPO, IL-1 α, p38 MAPK	Chandan et al. (2020)
Ginkgolides	Flavonoids, glycosides	*Ginkgo biloba*	Ginkgoaceae	MPO, TNF-α, IL-1β	Kotakadi et al. (2008), Mustafa et al. (2006)
Glycyrrhizin	Saponin	*Glycyrrhiza glabra*	Fabaceae	IL-6, IL-10, TNF-α, NO, NF-ĸB	Kim et al. (2006)
Gymnemic acid	Flavonoids	*Gymnema sylvestre*	Asclepiadaceae	TNF-α, SOD, CAT	Aleisa et al. (2014), Arun et al. (2014), David and Sudarsanam (2013)
Lonicerin	Flavonoid	*Lonicera japonica*	Caprifoliaceae	IL-1β, TNF-α, IL-6, IFN-γ, IL-12, IL-17	Lv et al. (2021), Shang et al. (2011), Zhou et al. (2021)
4-Methyl esculetin	Coumarin	*Scopolia carniolica*	Solanaceae	IL-1β, TNF α, IFN-γ, IL-2, IL-8	Witaicenis et al. (2012)
Rheum tanguticum polysaccharide	Polysaccharide	*Rheum tanguticum*	Polygonaceae	NF-kβ, Th1/Th2	Liu et al. (2003), Liu et al. (2005), Liu et al. (2008), Liu et al. (2009)
Shagoal	Phenol	*Zingiber officinale*	Zingiberaceae	NF-kβ, TNF-α, IL-1 β	Banerjee et al. (2011), Guo et al. (2021), Hsiang et al. (2013), Kumar Gupta and Sharma (2014)
Typhaneoside	Flavonoids	*Typha angustifolia*	Typhaceae	MPO, GSH, NO, SOD	Chen et al. (2017), Fruet et al. (2012)

*CAT* catalase, *COX-2* cycloxegenase-2, *GSH* glutathione, *IFN-γ* interferon-gamma, *IL* interleukin, *LOX-5* lipoxegenase-5, *LTB*_*4*_ leukotriene B4, *p38 MAPK* P-38 mitogen-activated protein kinases, *MCP-1* monocyte chemo attractant protein-1, *MIP-2* macrophage inflammatory protein-2, *MPO* myeloperoxidase, *NF-kβ* nuclear factor-Kappa β, *NO* nitric oxide, *PGE*_*2*_ prostaglandin E_2_, *ROS* reactive oxygen species, *Th1* type-1 T helper, *Th2* type-2 T helper, *SOD* superoxide dismutase, *TNF-α* tumor necrosis factor-alpha

Clinical data has depicted that patients with UC may have deficiency of many micronutrients such as vitamins and minerals due to loss of appetite, reduced absorption by the colon, and colonic diarrhea, and/or maybe due to different types of medication therapies. So, it is essential to overcome these deficiency states for which different kinds of nutraceuticals can be used. The role of probiotics in UC has been explored, and it has been documented that these agents can be used alone or in combination with other anti-ulcer agents.

Different probiotics such as *Lactobacillus salivarius*, *Lactobacillus acidophilus,* and *Bifidobacterium bifidum* along with mesalazine have been administered to UC patients for two years, and the response was evaluated according to the Modified Mayo Disease Activity Index. So, probiotics can help to avoid long-term use of corticosteroids in mild to moderate UC and can be used for induction of remission (Valdovinos et al. 2017; Palumbo et al. 2016; Mallon et al. 2006; Shigemori and Shimosato 2017; Hevia et al. 2015).

The role of *Escherichia coli *Nissle (*E. coli*/EcN) in patients suffering from UC has been demonstrated, and it has been reported that *E.coli* is effective and safe in maintaining remission in patients suffering from UC (Gallo et al. 2016; Fábrega et al. 2017; Scaldaferri et al. 2016).

A non-comparative clinical trial using a combination of probiotics (*Bifidobacterium, lactobacillus*, and *streptococcus*), commonly known as VSL#3, has been carried out, and down expression of toll like receptor (TLR) 2 and TLR4 was observed. Further, the intestinal epithelial up-regulation of protective IL-10 and down-regulation of IL-12, IL-17 and IL-23 has been achieved, which indicated its role in treatment of UC (Yao et al. 2017; Zhang et al. 2016).

The impact of polysaccharides from *Chrysanthemum morifolium* Ramat on the gut microbiota was assessed in ulcerative rats. Physiological investigations recommended that *Chrysanthemum* polysaccharides had quite defensive consequences for UC. It decreases the level of pro-inflammatory cytokines (such as IL-23, IL-6, TNF-α, and IFN-λ) and increases the level of anti-inflammatory mediators (like IL-4, IL-10, IL-11), thus re-establishing the state of eubiosis and restoring the immune system (Yao et al. 2017).

The role of *B. subtilis* has been evaluated in DSS-induced UC in mice, and its efficacy has been assessed by performing alcian blue staining, cytokine level by enzyme linked immunosorbent assay (ELISA), and microbiota composition. The effect is achieved by mucosal repairing and microbiota balance. *Lactobacillus rhamnosus* derived soluble protein acts by increasing mucus production in colonic epithelium. It causes thickening of mucus layer by modulating EGF factor (Zhang et al. 2016; Sun et al. 2016; Chapman et al. 2007).

Although many trials have been carried with phytoconstituents for UC (Table 3) but they were not able to establish much clinical efficacy due to a lack of data comparison with standard drugs. Hence, more studies need to be carried out to assess the role of natural compounds in UC. Along with the same, the safety profile of herbal products should also be done to determine toxic reactions and should be compared with conventional drugs. Moreover, the identification of active moieties in such products should be done to identify the new lead molecule.

**Table 3:** Clinical trials associated with UC

Clinical trial ID	Study title	Study start/completion date	Country	Phase	Phyto-constituent used	Condition	Type of formulation	Intervention	Summary	References
NCT02962245	Efficacy of treatment with berberine to maintain remission in ulcerative colitis	November 2016/January 2018	–	IV	Berberine	UC	–	Drug: Regular treatment with oral berberine 300 mg three times daily until recurrence in one year	The efficacy of berberine on reduction of the annual recurrence rate of UC is estimated	https://www.clinicaltrials.gov/ct2/show/record/NCT02962245
NCT01783119	Effect of *Aloe vera* in the inflammation of patients with mild ulcerative colitis	August 2012/December 2013	National Institute of Medical Science and Nutrition, Salvador, Zubirán D.f., Tlalpan, Mexico	I	Aloe vera	UC	Gel	Drug: Dietary Supplement: *Aloe barbadensis* Miller Consume 200 ml of aloe vera gel per day over a period of 3 months	Measuring the effect of the consumption of 200 ml of aloe vera gel daily for a period of 3 months reduces the degree of inflammation in patients with mild UC	https://www.clinicaltrials.gov/ct2/show/record/NCT01783119
NCT00578799	Effects of probiotics in patients with ulcerative colitis	December 2007/December 2007	University of California, Irvine, Health Sciences Medical Center, Orange, California, United States	I	–	UC	Capsule	Drug: Dietary Supplement: Kyo-Dophilus 5 × 10^9^ bacteria/capsule, twice a day, 1 in the morning, 1 in the evening is used	The effect of dietary supplement (5 × 10^9^ bacteria/capsule, twice a day) for 6 weeks in patients suffering from UC is estimated	https://www.clinicaltrials.gov/ct2/show/record/NCT00578799
NCT04223479	Effect of probiotic supplementation on the immune system in patients with ulcerative colitis in Amman, Jordan	January 2020/ongoing	Jordan University Hospital, Amman, Jordan	II		UC	Capsule	Drug: Administration of oral viable capsules of probiotic containing lactobacillus and bifidobacteria 3 times a per day for 2 weeks	The effect of using probiotics as an adjunct to medical therapy and its effect on the response of inflammatory markers, immune response, and quality of life is estimated	https://www.clinicaltrials.gov/ct2/show/record/NCT04223479
NCT04000139	Anthocyanin Rich Extract (ACRE) in patients with ulcerative colitis	April 2019/ongoing	Universitätsspital Basel, Basel, Switzerland Inselspital Bern, Bern, Switzerland Gastroenterologische Praxis Balsiger, Seibold & Partner, Bern, Switzerland	II		UC	Extract	Drug: Take 3 g of anthocyanin-rich extract daily as: 3 doses of 2 × 500 mg. Treatment duration 56 days (8 weeks)	The efficacy of anthocyanin rich extract is estimated in patients with UC	https://www.clinicaltrials.gov/ct2/show/record/NCT04000139
NCT01320436	Randomized, double-blind, placebo-controlled study to evaluated the efficacy of combining curcumin + 5ASA medication versus 5ASA medication alone on active mild to moderate ulcerative ccolitis patients	July 2011/September 2014	Sheba Medical Center, Ramat Gan, Israel	III	Curcumin	UC	Capsule	Drug: Take curcumin 3 capsules (820 mg containing 500 mg curcumin each) twice daily and 5-ASA according to clinical guidelines (4gr' per os + topical 1gr) mesalamine	The data provide bases for investigating an integrative approach to optimize the current standard treatment in UC patients	https://www.clinicaltrials.gov/ct2/show/record/NCT01320436
NCT03798210	Effect of *Lactobacillus reuteri* ATCC PTA 4659 in patients with ulcerative colitis	January 2017/31 January 2019	Uppsala University, Uppsala, Sweden	II		UC	–	Drug: Take dietary supplement: *Lactobacillus reuteri*	Investigation of the effect of the endogenous bacterium *Lactobacillus reuteri* ATCC PTA 4659 as a nutrient additive against relapse in UC is performed	https://www.clinicaltrials.gov/ct2/show/record/NCT03798210
NCT01479660	Role of healthy bacteria in ulcerative colitis	March 2011/October 2014	Post Graduate Institute of Medical Education and Research, Chandigarh, India	IV		UC	Capsule	Drug: Take probiotic capsules (450 billion CFU) orally daily for a period of 12 weeks and probiotic in higher dose of (3600 billion CFU) can be administered daily for a period of 12 weeks	The efficacy of probiotic for the restoration of intestinal permeability and reduction of intestinal inflammation in active UC can be estimate	https://www.clinicaltrials.gov/ct2/show/record/NCT01479660
NCT02488954	Interest of *Propionibacterium freudenreichii* for the treatment of mild to moderate ulcerative colitis	February 2016/Terminated	CHU de Rennes, Rennes, France	–	–	UC	–	Drug: Oral daily intake of probiotics in the form of cheese portion (50 g) during 8 weeks	Determine the role of *Propionibacterium freudenreichii* as anti-inflammatory agent in decreasing disease activity during UC	https://www.clinicaltrials.gov/ct2/show/record/NCT02488954
NCT02277223	Curcumin in paediatric inflammatory bowel disease	March 2020/Ongoing	Schneider Medical Center, Petach Tikva, Israel	III	Curcumin	UC	Capsule	Drug: Dietary supplement: curcumin, in addition to induction therapy, patients receive oral capsules of curcumin (Bara Herbs Inc): Weight < 20 kg: 1 g, twice daily, 20–30 kg: 1.5 g twice daily, weight > 30 kg: 2 g twice daily. For Maintenance, in addition to oral 5-ASA maintenance treatment, responding patients receive oral capsules of curcumin (Bara Herbs Inc): Weight < 30 kg: 500 mg, twice daily, weight > 30 kg: 1 g twice daily	Study helps to assess the efficacy of concomitant curcumin maintenance therapy for induction and maintenance therapy in paediatric UC patients	https://www.clinicaltrials.gov/ct2/show/record/NCT02277223
NCT04057547	Efficacy and safety of modified *Gegen qinlian* decoction for ulcerative colitis with damp-heat syndrome	April 2019/July 2019	Xiyuanhospital, Beijing, Beijing, China	I	–	UC	Decoction	Drug: Modified *Gegen qinlian* decoction containing *Pueraria lobata* 24 g, *Scutellaria baicalensis* 9 g, *Coptis chinensis* 9 g, artillery ginger 9 g, talc 9 g, roasted licorice 6 g, and granules is given	The efficacy of modified *Gegen qinlian* decoction in treatment of UC can be evaluated	https://www.clinicaltrials.gov/ct2/show/record/NCT04057547
NCT03565939	Probiotic treatment of ulcerative colitis with *Trichuris suis* Ova (TSO)	May 2018/on-going	Hvidovre Hospital, Hvidovre, Denmark	II	–	UC	–	Biological: *Trichuris suis* ova, eggs from the pig whipworm can be taken in treatment of UC	The study helps to achieve clinically meaningful responses in UC	https://www.clinicaltrials.gov/ct2/show/record/NCT03565939
NCT02683759	Bio-enhanced curcumin as an add-on treatment in maintaining remission of ulcerative colitis	February 2016/February 2017	Asian Institutes of Gastroenterology, Hyderabad, Telangana, India	III	Curcumin	UC	Capsule	Drug: Dietary supplement: bio-enhanced curcumin soft gelatin capsule Starting dose: 50 mg BID of bioenhanced curcumin increase dose to 100 mg after 2 weeks if there is no response	The potency of bio-enhanced curcumin soft gelatin capsule in tissue targeting and subsequently producing less adverse side effects can be evaluated	https://www.clinicaltrials.gov/ct2/show/record/NCT02683759
NCT02365480	Berberine chloride in preventing colorectal cancer in patients with ulcerative colitis in remission	June 2016/February 2018	Northwestern University Chicago, Illinois, United States Fourth Military Medical University Xi'an, Shaanxi, China	I	Berberine chloride	UC	–	Drug: Berberine chloride. Clinical efficacy of berberine chloride is measured using the UCDAI score	Safety of berberine (berberine chloride) administered to participants with UC in clinical remission can be assessed	https://www.clinicaltrials.gov/ct2/show/record/NCT02365480
NCT02683733	Bio-enhanced curcumin as an add-on treatment in mild to moderate ulcerative colitis	February 2016/February 2017	Asian Institutes of Gastroenterology, Hyderabad, Telangana, India	III	Curcumin	UC	Capsule	Drug: Take dietary supplement: bio-enhanced curcumin soft gelatin capsule for reemission in UC	Efficacy and tolerability of bio-enhanced curcumin (diferuloylmethane) in the induction of remission in patients with mild to moderate UC can be assessed	https://www.clinicaltrials.gov/ct2/show/record/NCT02683733
NCT02267694	Study of freeze-dried black raspberry in maintenance of ulcerative colitis	August 2013/October, 2015	University of Connecticut, Health Center, Farmington, Connecticut, United States	I	–	UC	Powder	Drug: Take freeze-dried black raspberry powder 5 g once daily for 4 weeks	The study helps to determine efficacy of raspberry in maintenance of remission of UC	https://www.clinicaltrials.gov/ct2/show/record/NCT02267694
NCT02442960	Evaluating safety and efficacy of herbal treatment in ulcerative colitis	December 2014/July, 2017	Stanford University, Palo Alto, California, United States	I	–	UC	Powder	Drug: Take herbal treatment of oral SA100 g twice daily for 8 weeks in patients suffering from UC	The study evaluates the safety and preliminary efficacy of oral SA100 in the treatment of patients with mild, moderate or severe UC	https://www.clinicaltrials.gov/ct2/show/record/NCT02442960
NCT00374725	Treatment of ulcerative colitis with a combination of *Lactobacillus rhamnosus* and *Lactobacillus acidophilus*	February 2003/not provided	Aarhus University Hospital, Aarhus, Denmark, Denmark	–	–	UC	–	Behavioral: Administration of probiotic (*L. rhamnosus* and *L. acidophilus*) in treatment of UC	The efficacy of combination of *Lactobacillus rhamnosus* and *Lactobacillus acidophilus* in UC patients can be evaluated	https://www.clinicaltrials.gov/ct2/show/record/NCT00374725
NCT00268164	*Lactobacillus acidophilus* and *Bifidobacterium animalis* Subsp. Lactis, maintenance treatment in ulcerative colitis	June 2004/March 2007	Dept. of Medical Gastroenterology, Hvidovre, Denmark	II	–	UC	–	Drug: Take lactic acid bacteria *Lactobacillus acidophilus* (LA5) and *Bifidobacterium animalis* subsp. lactis (BB12) for maintaining treatment in UC	Effectiveness of lactic acid bacteria *Lactobacillus acidophilus* (LA5) and *Bifidobacterium animalis* subsp. lactis in maintenance treatment in UC can be determined	https://www.clinicaltrials.gov/ct2/show/record/NCT00268164
NCT03415711	PRObiotic VSL#3^®^ for maintenance of clinical and endoscopic remission in ulcerative colitis	28 April 2017/24 April, 2019	Istituto di Medicina Interna CIC Columbus Policlinico Universitario Agostino Gemelli Università Cattolica del Sacro Cuore, Rome, Italy	–	–	UC	Sachets	Drug: Take dietary supplement: VSL#3^®^ 450 billion sachet once a day for maintaining remission in mild to moderate UC	Efficacy of VSL#3^®^ in the maintenance of clinical and endoscopic remission of mild-to-moderate UC can be determined	https://www.clinicaltrials.gov/ct2/show/record/NCT03415711
NCT00963287	Trial of Chinese prescription on ulcerative colitis	August 2009/July 2011	Longhua Hospital, Shanghai, Shanghai, China	–	–	UC	Decoction	Drug: basic prescription plus or minus herbs depend on symptoms, 2 times a day	Evaluation of the efficacy and safety of the Chinese prescription on UC can be performed	https://www.clinicaltrials.gov/ct2/show/record/NCT00963287
NCT04006977	Multistrain probiotics reduces UC depression and anxiety scores	October 2019/February 2020	Xijing Digestive Disease, Xi'an, Shaanxi, China	–	–	UC	Sachet	Dietary Supplement: receive standard medical therapy plus the multistrain probiotics (DSF), 4 sachets per day	Multistrain probiotic product (de simone formulation) reduces depression and anxiety scores in patients with UC	https://www.clinicaltrials.gov/ct2/show/record/NCT04006977
NCT04102852	*Lactobacillus rhamnosus* GG (ATCC 53103) in mild-moderately active UC patients	September 2019/ongoing	S. Giovanni Addolorata Hospital, Rome, Italy	I and II	–	UC	–	Dietary Supplement: *Lactobacillus rhamnosus* GG ATCC 53103 probiotic administration at two different doses for 1 month for UC	The role of *Lactobacillus rhamnosus* GG in the modulation of the inflammatory process in the mucosa of UC patients with mild-moderate clinical activity	https://www.clinicaltrials.gov/ct2/show/record/NCT04102852
NCT00510978	Probiotics in gastro intestinal disorders	January 2002/not provided	Cork University Hospital Cork, Co Cork, Ireland	II and III	–	UC, CD	Sachet	Biological: *Bifidobacterium infantis* 35624 1 sachet/day for one year Biological: *Lactobacillus salivarius* UCC118 1 sachet per day for 1 year can be taken	The efficacy of probiotics, *Bifidobacterium infantis* 35624 or *Lactobacillus salivarius*, as food supplements for maintenance of remission in CD and UC can be estimated	https://www.clinicaltrials.gov/ct2/show/NCT00510978
NCT04753775	Randomized, double-blind, placebo-controlled trial of enema aloe vera gel in active ulcerative proctosigmoiditis	March 2010/April 2010	–	–	–	UC	Gel	Drug: Aloe vera gel enema for achieving remission in active ulcerative proctosigmoiditis	The efficacy of Aloe vera gel formulation as topical therapy in active UC can be determined	https://www.clinicaltrials.gov/ct2/show/record/NCT04753775
NCT01037322	Cannabidiol for inflammatory bowel disease	January 2010/September 2012	Sapir Medical center Meir Hospital, Kefar Saba, Israel	I and II	Cannabidiol	UC, IBD	–	Drug: Cannabidiol in olive oil drops, 5 mg twice daily	The effect of cannabidiol on disease activity in patients with IBD is evaluated	https://www.clinicaltrials.gov/ct2/show/record/NCT01037322
NCT01765439	The Effect of VSL#3 probiotic preparation on the bile acid metabolism in patients with inflammatory bowel disease	February 2014/on-going	Istituto di Medicina Interna CIC Columbus Policlinico Universitario Agostino Gemelli Università Cattolica del Sacro Cuore, Roma, Italy	–	–	UC, IBD	Sachet	Dietary Supplement: VSL#3 (Original De Simone formulation) give patients two sachets of VSL#3 probiotic (i.e. 2 × 900 billions of live bacteria) per day (one in the morning, one in the evening) for 6 weeks	With the study, efficacy of administration of VSL#3 (Original De Simone formulation) probiotic preparation in patients with IBD can be determined	https://www.clinicaltrials.gov/ct2/show/record/NCT01765439
NCT01078935	The effect of probiotics on the rate of recovery of inflammatory bowel disease exacerbation, endothelial function, and markers of inflammation	December 2012/April 2014	–	IV	–	UC		Dietary supplement: Give probiotics medication for 6 weeks to patients suffering from UC	Study determines rate of recovery of IBD exacerbation, endothelial function, and markers of inflammation in patients with UC	https://www.clinicaltrials.gov/ct2/show/record/NCT01078935
NCT00889161	Curcumin in paediatric inflammatory bowel disease	May 2009/June 2010	Seattle Children's Hospital, Seattle, Washington, United States	I	Curcumin	UC, IBD, CD	–	Drug: Curcumin Give initial dose of 500 mg twice a day for 3 weeks followed by 1 g twice a day at Week 3 for a total of 3 weeks and then titrated again to 2 g twice a day at week 6 for 3 weeks	Appropriate dose of curcumin in paediatric patients with IBD is determined	https://www.clinicaltrials.gov/ct2/show/record/NCT00889161
NCT02735941	Study on cannabinoid receptor expression in gastrointestinal diseases	June 2017/July 27, 2018	Medical University of Graz, Graz, Austria	–	Cannabinoid	UC, CD, Colon cancer	–	Not provided	The study examines expression of cannabinoid receptors in mucosal biopsies of the colon and blood leukocytes of patients with IBD	https://www.clinicaltrials.gov/ct2/show/record/NCT02735941
NCT01496053	Anti-inflammatory effect of Agaricus Blazei Murill in inflammatory bowel disease (IBD)	December 2011/December 2015	Oslo University Hospital, Ulleval, Oslo, Norway	II and III		UC, IBD, CD	Extract	Dietary Supplement: Take AndoSan 30 mL × 2 for 21 days	Improvement in immunomodulatory effect of mushroom extract (AndoSanTM) in patients with UC and CD can be studied	https://www.clinicaltrials.gov/ct2/show/record/NCT01496053
NCT02227602	Anti-inflammatory effects of mango polyphenolics in inflammatory bowel disease	January 2014/May 2017	Texas A&M University, Clinical Lab, Nutrition and Food Science Department, College Station, Texas, United States	–		Intestinal disease, IBD, UC	–	Drug: Mango polyphenolics provide frozen mango pack (200–400 g per day)	The study determines whether mango consumption improves biomarkers for inflammation in IBD patients	https://www.clinicaltrials.gov/ct2/show/record/NCT02227602
NCT03266484	Effect of a probiotic mixture on the gut microbiome and fatigue in patients with quiescent inflammatory bowel disease	November 2017/ongoing	Crohn's and Colitis Center, MGH, Boston, Massachusetts, United States	–	–	IBD, CD, UC	–	Dietary Supplement: Probiotic supplement contains 8 different strains of bacteria and participants are dosed in two dosages per a total of 40 billion bacteria daily	Evaluation of effect of dietary therapy with a probiotic mixture on the gut microbiome and fatigue symptoms in patients with IBD can be assessed	https://www.clinicaltrials.gov/ct2/show/record/NCT03266484
NCT04749576	Saffron as anti inflammatory agent in patients with inflammatory bowel disease	15 December 2020/ongoing	Howard University Hospital, Washington, District of Columbia, United States	–	–	UC	–	Dietary Supplement: saffron supplement for IBD	Efficacy of nutritional saffron supplement as an anti inflammatory agent in patients with IBD is estimated	https://clinicaltrials.gov/ct2/show/record/NCT04749576
NCT02865707	Ulcerative colitis relapse prevention by prebiotics	August 2016/February 2020	University of Alberta, Edmonton, Alberta, Canada	–	–	UC	–	Dietary Supplement: Synergy-1, which is chicory-derived β-fructans inulin plus FOS (1:1)	Efficacy and preventive mechanism of prebiotics in UC can be estimated	https://clinicaltrials.gov/ct2/show/record/NCT02865707
NCT03000101	Study of the role of pomegranate juice ellagitannins in the modulation of inflammation in inflammatory bowel disease	January 2017/ongoing	U.O. Gastroenterologia-Azienda Ospedaliero-Universitaria di Bologna, Policlinico Sant'Orsola-Malpighi, Bologna, Italy	–	–	UC, CD	Juice	Other: 100% pomegranate juice 125 mL of 100% pomegranate juice twice daily for 12 weeks	The study investigates preventive effects of dietary phenolics in UC	https://clinicaltrials.gov/ct2/show/record/NCT03000101

*CD* Crohn’s disease, *IBD* inflammatory bowel disease, *UC* ulcerative colitis

## Safety concerns of herbal products used in UC

Although, a large number of herbal products have been explored for treatment of UC but safety profile of herbs should be considered for being used in human beings. A double blind, randomized and placebo controlled study of AV gel was performed to evaluate its efficacy and safety in patients suffering from mild to moderate colitis. Forty four out patients were randomly chosen and AV gel or placebo treatment twice daily was given. The protective effect of AV gel was assessed by primary (clinical, sigmoidoscopic and histological remission) and secondary (colitis activity index, Baron score, histology score, C-reactive protein) outcomes. Adverse effects reported by patients were minor and were not directly correlated with consumption of AV gel as the side effects such as bloating, foot pain, sore throat, and acne were also reported by patients on placebo treatment, which advocated AV gel as safe for curing UC (Langmead et al. 2004).

In another study, the sub-acute, acute and genotoxicity of *A. vera* soft gelatin capsules (ASC) were estimated in ICR male and female rats. The acute toxicity study was estimated at a dose of 15,000 mg/kg body weight, whereas for sub-acute study, the blended dose in range of 832.5 to 3330 mg/kg was used. No changes in body weight, behavior, biochemical, histopathalogical parameters and mortality were observed, which indicated that lethal dose of ASC is above 15,000 mg/kg. Genotoxicity of ASC was determined using Ames test (10,000 mg/kg) and no evidence of bone marrow micronucleus and testicular chromosome abnormality was found, hence can be considered safe on oral administration (Biancone et al. 2008).

In a recent study, the toxicity of AL fruit extract was determined in female Wistar rats using acute and repeated models. In acute toxicity study, the animals were administered two different doses i.e. 1000 and 5000 mg/kg, whereas for sub-acute toxicity study, 300 mg/kg dose was given for a period of 4 weeks. As, no mortality was observed in animals, hence can be considered as safe therapeutic option (Yaghoubi et al. 2019).

The safety profile of *B. serrata* extract and AKBA was demonstrated as no alteration in intestinal cell viability, barrier functions and integrity of biomarkers was observed; therefore, these can be used as a safe adjuvant for UC patients (Catanzaro et al. 2015). The double-blind placebo controlled randomized study was performed in 108 outpatients with CD and clinical remission and rate of relapse were determined after oral administration of *B. serrata* extract, Boswelan (3 × 2 capsule/day; 400 mg) for 52 days. The results indicated that *B. serrata* can be tolerated safely for treatment of IBD (Holtmeier et al. 2011).

No toxicity and mortality was observed in mice treated with different doses (700, 1400 and 2800 mg/kg) of *C. sinensis* extract, indicating safety profile of tea (Olayinka et al. 2018). Further, genotoxicity study of catechin was estimated by micronucleus and big blue transgenic rodent mutation assays in ICR mice after single or multiple oral administration of catechin preparation and lack of significant mutagenic and clastogenic concern confirmed its potential and safety in human beings (Ogura et al. 2008).

Chandan et al. (2020) has shown promising effect of curcumin in TNBS induced UC and it was observed that curcumin at a dose of 0.75–7.5 g/kg/day did not cause any abnormality in mice. Acute and sub-acute toxicity of *C. longa* extract was evaluated at dose 30–240 mg/kg in Wistar rats using Organization for Economic Co-Operation and Development (OECD) 425 and 407 guidelines, respectively. No risk of toxicity was observed at any selected dose of curcumin, which suggested the safety of curcumin (Kamsu et al. 2019).

A large number of studies have indicated non-toxic nature of glycyrrhizin as it neither exhibit teratogenic nor mutagenic effects and its daily recommended dose can be up to 0.015–0.229 mg/kg of the body weight (Isbrucker and Burdock 2006).

The acute toxicity of homeopathic preparation of Gurmur (*G. sylvestre*) was determined in Sprague Dawley rats. No significant difference was observed in haematological, biochemical and histopathological parameters of placebo and Gurmur treated animals. Further, no mortality was observed in animals and can be recommended at a dose of 300 mg/kg body weight safely (Shukla et al. 2020).

Further, safety assessment of fermented *Phylloporia ribis* (*Lonicera japonica* Thunb) was performed in Sprague–Dawley rats and no adverse effects were observed in animals in both acute and sub-chronic toxicity study, indicating its potential in treatment of UC (Lu et al. 2014).

Another double blind, randomized, controlled study indicating the efficacy of ginger capsule in UC patients was performed. The down-regulation of inflammatory mediators and high sensitivity of C-reactive protein (hs-CRP) showed protective effect in treatment of UC and can be given up to 3 g/kg (Shayesteh et al. 2020).

Various reported studies have demonstrated that herbal products are an effective and safe option for treatment of UC.

## Marketed herbal formulations for ulcerative colitis

A large number of herbal products such as Kutajghan vati, Vatsakadi churna, Arjuna capsule, and pitta balance capsule are mainly available in Indian market for the treatment of UC.

Kutajghan vati containing Kutaj (*Holarrhena antidysenterica*) is prescribed in dose of two tablets of 250 mg, twice a day. It is manufactured by various Indian pharmaceutical companies including Patanajali Ayurved Ltd., Haridwar (Uttarakhand) (Patanjali 2021), Baidyanath Ayurved Bhavan (Pvt) Ltd., Jhansi (Uttar Pradesh) (Baidyanath 2021).

Vatsakadi churna composed of Kutaj (*H. antidysenterica*), Bilva (*Aegle marmelos*), and Saunf (*Foeniculum vulgare*) is used at a dose of one tablespoonful (3–6 g) twice a day. It is manufactured by pharmaceutical company Planet Ayurveda (Planet Ayurveda 2021a, b).

Arjuna capsule/tablet containing active constituent of Arjuna (*Terminalia arjuna*) is prescribed as two capsules of 500 mg each twice a day. It is manufactured by various Indian pharmaceutical companies like The Himalaya Drug Company (Himalaya 2021), Indian Herbo Pharma (Indian Herbopharma 2021), and Sona Health care (Sona Health Care 2021).

Pitta balance capsule prepared by pharmaceutical company Planet Ayurveda, is a very effective anti-ulcerogenic herbal preparation containing Praval pishti (coral calcium), Akik pishti (agate calcium), Jawar mohar pishti (calcium compound), Kamdudha rasa (calcium compound), Mukta pishti (pearl calcium), and Giloy satva (*Tinospora cordifolia*). The recommended therapeutic dose of Pitta balance is one capsule of 675 mg twice a day (Planet Ayurveda 2021a, b).

## Concluding remarks and future perspectives

Although numerous conventional and non-conventional treatment options are available for UC, all of these suffer from various drawbacks such as safety, efficacy, and high cost. Usually, the therapy of UC requires treatment and maintenance of reemission for the entire life period, so these side effects assume much more significance. Herbal products are alternative medicines used to relieve UC with much milder side effects as compared to those associated with the present medicine system. According to a study conducted by WHO, 80–85% of the world population relies on plant-derived products that offer much promise for the treatment of UC but still require further investigation in preclinical and clinical fields to prove their safety, efficacy, and usefulness.

## Data Availability

Enquiries about data availability should be directed to the authors.

## Abbreviations

AA: Acetic acid
CAT: Catalase
CD: Crohn’s disease
COX-2: Cycloxegenase-2
DAI: Disease activity index
DNBS: Dinitrobenzene sulfonic acid
DSS: Dextran sodium sulphate
GSH: Glutathione
IBD: Inflammatory bowel disease
ICAM: Intercellular adhesion molecule
iNOS: Inducible nitric oxide synthase
IFN: Interferon
IL: Interleukin
JAK: Janus kinase
LOX: Lipoxygenase
LPS: Lipopolysaccharide
LT: Leukotriene
MadCAM: Mucosal vascular addressin cell adhesion molecule
MAPK: Mitogen-activated protein kinase
MCP: Monocyte chemoattractant protein
MDA: Malondialdehyde
MIP: Macrophage inflammatory protein
MMP: Matrix metalloproteinase
MPO: Myeloperoxidase
mRNA: Messenger ribonucleic acid
NF-κβ: Nuclear factor-kappa β
p38MAPK: P-38 mitogen-activated protein kinase
NO: Nitric oxide
PG: Prostaglandin
ROS: Reactive oxygen species
RNS: Reactive nitrogen species
SOD: Superoxide dismutase
TBARS: Thiobarbituric acid reactive species
TGF: Transforming growth factor
Th: T helper
TNBS: 2, 4, 6-Trinitrobenzene sulfonic acid
TLR: Toll-like receptor
TNF-α: Tumor necrosis factor-alpha
UC: Ulcerative colitis
VCAM: Vascular cell adhesion protein
WHO: World Health Organization
